# Reducing PI4KIIIα Levels or Activity Limits Tau Seed Internalization and Assembly in Human Cortical Neurons

**DOI:** 10.3390/cells15131228

**Published:** 2026-07-07

**Authors:** Eleonora Clemente, Ramakrishnan Sivasubramanian, Susanne Kordes, Priyanka Bhatia, Ruth Hans, Stefanie Vogel, Matthias Baumann, Bert Klebl, Jared Sterneckert

**Affiliations:** 1 Center for Regenerative Therapies TU Dresden (CRTD), Technische Universität Dresden, 01307 Dresden, Germany; 2 Lead Discovery Center GmbH, 44227 Dortmund, Germany; 3 Max Delbrück Center, 13125 Berlin, Germany; 4 Light Microscopy Facility, Center for Molecular and Cellular Bioengineering (CMCB), Technische Universität Dresden, 01307 Dresden, Germany; 5 Physics of Life, Technische Universität Dresden, 01307 Dresden, Germany; 6 Medical Faculty Carl Gustav Carus of TU Dresden, 01307 Dresden, Germany

**Keywords:** tauopathy, prion-like spreading, PI4KA, tau, PI4KIIIα, induced pluripotent stem cells, iPS cells, disease modeling

## Abstract

**Highlights:**

**What are the main findings?**

- PI4KIIIα regulates tau seed-induced pathology in biosensor cells and iPS cell-derived cortical neurons.
- PI4KIIIα regulates the intracellular levels of tau seeds.

**What are the implications of the main findings?**

- PI4KIIIα is a small-molecule-accessible target that may contribute to spreading of tau pathology.

**Abstract:**

Tau protein aggregation and spreading are central features of neurodegenerative diseases such as Alzheimer’s disease and frontotemporal dementia. Here, we investigated the role of phosphatidylinositol 4-kinase type IIIα (PI4KIIIα) in regulating tau propagation. We first used tau biosensor cells to demonstrate that both pharmacological inhibition and genetic reduction in PI4KIIIα effectively reduce the seeding of tau aggregation by extracellular seeds. To extend these findings to a more physiologically relevant system, we generated induced pluripotent stem (iPS) cell-derived cortical neurons carrying pathogenic *MAPT* mutations. These neurons rapidly acquired tauopathy-associated features, including expression of disease-relevant isoforms such as 4R tau, thereby enabling in vitro modeling of tau pathology. Using this model, we established phenotypic assays to monitor tau propagation and aggregation and applied them to test candidate small molecules. Notably, inhibition of PI4KIIIα consistently reduced seeding of tau assemblies in human neurons, highlighting this kinase as an important player in the seeding of tau pathology. Collectively, our work identifies PI4KIIIα as a regulator of tau pathology and provides new experimental platforms to dissect the molecular mechanisms of tau propagation. These findings open potential avenues for the development of strategies to slow or prevent tau-mediated neurodegeneration in the central nervous system.

## 1. Introduction

Tauopathies are one of the most common classes of neurodegenerative diseases. The term tauopathy refers to all diseases characterized by insoluble aggregates of tau protein, a microtubule-binding protein encoded by the *MAPT* gene, observed in the brain at the time of autopsy. Tauopathies affect a substantial number of people globally. They encompass several conditions, each of them with a different prevalence and population impact. Up to 26 different types of tauopathy have been identified [1]. Clinical manifestations of these diseases include memory, cognitive and behavioral disorders, such as dementia, as well as motoric dysfunction and parkinsonism [2].

When exposing tau monomers to pre-formed tau seeds in vitro, the latter can recruit tau monomers in suspension and further elongate the seed or generate new assemblies. The final tau fibrils resemble the original ones [3] leading to the speculation that different tau conformations or strains could self-replicate themselves. This idea is also known as “templated misfolding”. Pathological tau seeds may spread between neurons in a prion-like manner, contributing to the clinical progression of tauopathy phenotypes. In support of this idea, the group of Marc Diamond generated tau biosensor cells using human embryonic kidney 293 (HEK293) cells transgenically expressing fluorescently tagged tau repeat domain (tauRD). Purified tau fibrils as well as patient-derived brain homogenate caused aggregation of the tauRD reporter [4,5], which is consistent with the idea of pathological tau spreading.

Transgenic mouse lines provide evidence in support of tauopathy spreading in vivo. The Virginia Lee group developed two of the most widely used mouse models to study tau spreading. They generated a transgenic mouse expressing human tau, wild type (WT) and a second one harboring the tauopathy associated mutation P301S (PS19 mice). These PS19 mice showed fibrillary aggregates at 6 months of age, as well as progressive pathology spreading to the hippocampus and entorhinal cortex at the age of 12 months, accompanied by neuronal loss [6]. Cerebral injection of brain extract of PS19 mice with tau pathology into human WT-tau protein expressing mice induced pathology and spreading of tauopathy to brain connected regions from the site of injection [7]. The spreading of aggregation was caused by tau insoluble fractions, suggesting that insoluble tau species might be responsible for seeded aggregation [7]. Importantly, human WT-tau protein expressing mice did not show neurodegeneration whereas the PS19 mice did, suggesting that the sequence of tau protein being expressed plays an important role in aggregation and neurodegeneration [7]. These studies suggested that specific tau assemblies might have the ability to enter cells from the extracellular space and induce pathology, thereby spreading from cell to cell.

Internalization of exogenous tau seeds by neurons is an important step in the progression of tauopathies. Endocytosis contributes to tau seed internalization [8], and phosphatidylinositol 4,5-bisphosphate (PI(4,5)P_2_) plays an important role in clathrin-mediated endocytosis [9] as well as clathrin-independent endocytosis [10]. PI(4,5)P_2_ is formed by a two-step process. First, phosphatidylinositol is phosphorylated by phosphatidylinositol 4-kinase, including PI4KIIIα, to form phosphatidylinositol 4-phosphosphate (PI(4)P), which is subsequently phosphorylated to form PI(4,5)P_2_. Inhibition of PI4KIIIα has been shown to inhibit entry and replication of multiple exogenous pathogens, including Ebola virus [11,12] and Hepatitis C virus [13,14]. Consequently, pharmaceutical companies have shown interest in developing selective inhibitors targeting these kinases, which has led to the discovery of the potent inhibitors GSK-A1 and GSK-F1 [15].

Here, we sought to test the hypothesis that PI4KIIIα regulates the formation of tau assemblies after exposure to exogenous tau seeds. We demonstrate that multiple small-molecule inhibitors of PI4KIIIα reduced tau seeding in tau biosensor cells, which express TauRD P301S, and the inhibition of PI4KIIIα reduced the intracellular levels of exogenous tau seeds. To complement these results, we generated a human model tau pathology using induced pluripotent stem (iPS) cell-derived neurons. We demonstrate that GSK-A1, the most potent PI4KIIIα inhibitor tested, effectively reduced the intracellular levels of exogenous tau seeds as well as seed-induced formation of tau assemblies in cortical neurons differentiated from iPS cells with two MAPT mutations: P301S as well as IVS10+16. These findings are consistent with the hypothesis that PI4KIIIα regulates the internalization of exogenous tau seeds, but additional experiments would be needed to address other possible mechanisms such as intracellular processing of internalized tau seeds as well as regulation of endogenous tau levels.

## 2. Materials and Methods

### 2.1. Expression, Purification, Seeds Production and Labeling of Tau Variants

Tau K18(WT) and K18(P301L) were expressed and purified from insect cells. Briefly, tau K18(WT) and K18(P301L) (amino acids Q244–E372) were expressed in Hi5 insect cells using P3 baculovirus at a 1:67 ratio in 2 L total volume, cultured at 28 °C and 100 rpm, and harvested 48 h post-infection. Cells were lysed in HEPES-based buffer with Benzonase, Triton X-100, and protease inhibitors, then sonicated and centrifuged. The soluble fraction was purified via Ni-NTA affinity chromatography, followed by overnight dialysis and protease cleavage. The sample was concentrated and purified by size exclusion chromatography (S75) and MBP-tag removal. Final pure K18 protein was aliquoted and stored at –80 °C.

Full length tau protein (hTau441, 2N4R) was generated by the EMBL Protein Expression and Purification Core Facility (Heidelberg, Germany). Briefly, hTau441 was expressed in 2 L Sf21 insect cells at 1 × 10^6^ cells/mL using P1 virus at a 1:400 ratio, with 72 h expression. The cell pellet was resuspended in 80 mL lysis buffer supplemented with 10 mM magnesium chloride, benzonase, and EDTA-free protease inhibitors. Cells were lysed using a microfluidizer (3 passes) and lysates were centrifuged at 40,000 rpm for 30 min. The supernatant was boiled at 100 °C for 30 min, centrifuged again under the same conditions, and the final supernatant was applied to tandem 5 mL HiTrap Q and HiTrap SP columns. hTau441 was eluted from the HiTrap SP column using a 60 min gradient. Eluted fractions were pooled, concentrated, and further purified by size exclusion chromatography (Superdex 200 16/600). Final purified hTau441 was concentrated, aliquoted (100 µL), flash-frozen in liquid nitrogen, and stored at –80 °C.

### 2.2. HEK293T and Tau Biosensor Cells

TauRD P301S FRET Biosensor cells were purchased by ATCC (Manassas, VA, USA) (Catalog number CRL-3275). HEK293T and tau biosensors cells were maintained in DMEM with 10% fetal bovine serum, 1% l-glutamine, and 1% pen/strep. Media was replaced every 2–3 days, and cells were passaged once or twice a week using trypsin depending on their confluence.

### 2.3. Seeding Assays on Tau Biosensor Cells and Compound Testing

Tau biosensor cells were dissociated using Trypsin-EDTA and plated on gelatin-coated 96-well multiwell plates at a cell density of 30,000–35,000 cells per well. The day after, cells were treated with different compound concentrations or a volume of DMSO as mina control equal to the highest compound concentration tested. After 1 h of treatment (unless otherwise specified in the results section), tau K18 seeds were transfected using 1 μL of Lipofectamine^TM^ 2000 (Thermo Fisher Scientific, Waltham, MA, USA) per well. Tau seeds final concentration was always 250 nM. Twenty-four hours after transfection, cells were fixed with 4% paraformaldehyde (PFA) for 15 min. PFA was removed, and nuclei were stained overnight with 10 µg/mL Hoechst 33342 in DPBS. The day after, Hoechst 33342 solution was exchanged to fresh DPBS and cells were stored at 4 °C and protected from light until imaging.

Seeding assays were imaged using a Zeiss Apotome 1 inverted microscope (Zeiss, Oberkochen, Germany) using the tiles scanning option to avoid possible observation bias. Treatments were performed in three technical replicates for each individual experiment, and ten images were taken for each tested well, resulting in 30 images per each tested condition of each independent replicate. The tauRD CFP/YFP reporter was imaged using the GFP filter, that can detect both fluorophores.

Image analysis was performed using ImageJ software version 2.14.0. Fold change in aggregates/cell was calculated by dividing each value by the average DMSO value. Statistical significance was calculated by pooling the data points from all independent experiments. Each experiment was performed a minimum of three times, unless otherwise specified. Samples were tested for normality to choose the appropriate statistical test. The statistical test used for each experiment is specified in the figure legend for each graph.

### 2.4. PI4KA Knockdown and Seeding Assay on Tau Biosensor Cells

Tau biosensor cells were seeded at a density of 1 × 10^6^ cells per well in 6-well multiwell plates for each experimental condition. Cells were transfected with either a scrambled non-targeting siRNA or an on-target siRNA targeting *PI4KA*, which encodes PI4KIIIα protein, using 12.5 µL of Lipofectamine™ 2000 per well, at a final siRNA concentration of 100 nM. A mock control containing Lipofectamine-only (no siRNA) was included in each experiment to calculate relative protein expression fold changes. The transfection complexes were incubated at room temperature for 20 min before being added dropwise to the wells. The following day, the culture medium was replaced with HEK complete medium supplemented with 2% FBS to reduce cell proliferation. Cell lysates were collected 48 h post-transfection, and PI4KIIIα protein levels were analyzed by Western blot, normalized to the endogenous control protein glyceraldehyde 3-phosphate dehydrogenase (GAPDH).

For aggregation assays, tau biosensor cells were dissociated using Trypsin-EDTA, and seeded at a density of 30,000 cells per well on Cultrex-RGF-coated 96-well multiwell plates. Twenty-four hours after plating the cells, they were transfected with either scrambled or *PI4KA*-targeting siRNA at a final concentration of 100 nM, using 1.25 µL of Lipofectamine™ 2000 per well. The next day, the medium was replaced with HEK complete medium containing 2% FBS. Forty-eight hours post-siRNA transfection, tau K18 seeds were transfected at a final concentration of 250 nM using 1 µL of Lipofectamine™ 2000 per well. Twenty-four hours after seed transfection, cells were fixed with 4% PFA for 15 min. Following fixation, the PFA was removed, and cells were incubated overnight with 10 µg/mL Hoechst 33342 in DPBS. Samples were then stored in DPBS until imaging.

To evaluate tauRD-CFP reporter expression, siRNAs were transfected into tau biosensor cells seeded on 96-well plates under the same conditions described above. Cells were fixed with 4% PFA for 15 min, followed by overnight incubation with 10 µg/mL Hoechst 33342 in DPBS. The next day, the staining solution was replaced with fresh DPBS, and samples were stored at 4 °C until imaging. All conditions were performed in technical triplicates. Whole-cell volumes were imaged using an LSM780 laser scanning confocal microscope (Zeiss, Oberkochen, Germany). Maximum intensity projections were generated, and CFP fluorescence intensity was quantified using ImageJ.

### 2.5. FL-Tau-488 Internalization Assay on HEK293T

HEK293T cells were seeded at a density of 20,000 cells per well in 96-well plates. The following day, cells were treated for 1 h with either vehicle control (DMSO) or GSK-F1 at a 1 μM final concentration. FL-tau-488 seeds were transfected using 1 µL of Lipofectamine™ 2000 per well at a final concentration of 100 nM. Six hours post-transfection, cells were incubated with Wheat Germ Agglutinin (WGA)-Alexa Fluor™ 594 (Thermo Fisher Scientific, Waltham, MA, USA) at a 3.3 μg/mL final concentration for 30 min to label the cell membrane. Cells were then washed three times with DPBS, followed by fixation with 4% PFA for 15 min at room temperature. Fixed cells were incubated overnight with 10 µg/mL Hoechst 33342 in DPBS, and later stored at 4 °C until image acquisition.

Plates were imaged in collaboration with the Technology Development Studio (TDS) facility at the Max Planck Institute of Molecular Cell Biology and Genetics (MPI-CBG, Dresden, Germany) in an automated manner (in order to reduce observation bias) using a Cell Voyager CV7000 high-content imaging system (Yokogawa, Tokyo, Japan) at 60× magnification. Each experimental condition was tested in three technical replicates, and nine images per well were acquired, resulting in a total of 27 images per condition and biological replicate. Maximum intensity projections were generated, and FL-tau-488 puncta were quantified by segmenting nuclei and WGA-stained areas using ImageJ software. Fold change in tau puncta per cell was calculated by dividing each test condition value by the average value of the DMSO control. Statistical significance was calculated by pooling all data points from independent experiments. Samples were tested for normality to choose the appropriate statistical test. The statistical test used for each experiment is specified in the figure legend for each graph.

### 2.6. iPS Cell Culture and Generation of NGN2-Inducible Lines

iPS cells *MAPT*-WT originally available from Sigma-Aldrich (Burlington, MA, USA, catalog number iPSC0028) and discontinued by the company, were transferred from the laboratory of Prof. Marcel Leist (University of Konstanz) with Sigma-Aldrich’s approval. iPS cells *MAPT*-Mut (SIGi001-A-9) were purchased through European Bank for induced pluripotent Stem Cells (EBiSC). These cell lines were generated using a protocol and informed consent approved by the local ethics commission [16].

All iPS cell lines were maintained at 37 °C and 5% CO_2_ in mTeSR^TM^ 1 medium on Cultrex-SC matrix to promote colony growth. Cells were passaged every 5–7 days using a clump passaging method with dispase. Briefly, differentiated areas were manually removed, and cells were washed twice with DMEM/F-12 before incubation with 1 mL dispase for 5–10 min at 37 °C. Following visual assessment of detachment, the dispase was aspirated, and cells were washed twice with DMEM/F-12. Colonies were then collected using a cell scraper, transferred to a centrifuge tube, and spun at 300× *g* for 5 min. After removing the supernatant, the pellet was gently resuspended in 1 mL mTeSR^TM^ 1 (Stem Cell Technologies, Vancouver, Canada) medium, mechanically dissociated into the desired clump size, and replated in 6-well multiwell plates at the appropriate dilution.

For cryopreservation, the same procedure was followed until centrifugation. The cell pellet was then resuspended in cryopreservation medium (10% DMSO in mTeSR^TM^ 1) and transferred to cryovials. Cryovials were placed in a Mr. Frosty^TM^ freezing container (Sigma-Aldrich, Burlington, MA, USA) at −80 °C overnight and subsequently transferred to liquid nitrogen storage. Cells were typically frozen when one well of a 6-well plate reached 70–80% confluency, with the content of one well stored per cryovial.

For genome editing, iPS cells were passaged using dispase and seeded into 12-well plates. Transfection was carried out using 2.5 µg of the pUCM-NGN2 donor vector and 2.5 µg of the pXAT2 vector, which encodes Cas9 and the guide RNA (gRNA) targeting the NGN2 locus. Both plasmids were mixed with 10 µL of Lipofectamine^TM^ Stem Transfection Reagent (Thermo Fisher Scientific, Waltham, MA, USA) and 250 µL of Opti-MEM^TM^, incubated for 10 min at room temperature, and then added to the cells. The culture medium was replaced one day post-transfection. Puromycin selection was initiated 72 h after transfection at a final concentration of 0.4 µg/mL, and was refreshed daily for a total of 10 days. Following selection, cells were maintained as bulk cultures and passaged twice before being dissociated into single cells for clonal isolation. Between 5 and 10 individual clones per cell line were manually picked and screened by PCR to confirm the correct insertion of the NGN2 doxycycline-inducible transgene. A single positive clone per cell line was selected for downstream experiments and subjected to G-banding karyotyping to confirm genomic integrity following editing.

### 2.7. NGN2-Induced Neuronal Differentiation

When iPS-cells reached 70–80% confluency, cells were dissociated using accutase, and then counted and seeded at a final density of 50,000–100,000 cells/cm^2^ onto cultrex-RGF pre-coated plates. For general purposes, differentiation was performed on 6-well multiwell plates or scaled up to P100 Petri dishes in order to produce batches of neurons for cryopreservation. On the day of dissociation, cells were plated in induction media supplemented with 10 µM Rock inhibitor and 2 µg/mL doxycycline. On day 2 and 3 of differentiation, media was replaced with fresh induction media supplemented with 0.2 µM Compound E and 2 µg/mL doxycycline. Small molecules were added to the media directly before use. On day 4, media was replaced with maturation media supplemented with 4 µM 5-FdU to eliminate remaining proliferative cells. On day 5, cells already displayed neuron-like morphology with short neurites, and were dissociated with accutase, counted and plated onto PLO-Laminin pre-coated plates. For immunofluorescence and cellular assays 50,000 cells/well were plated in 96-well plates or 10^6^ cells/well in 12-well plates for protein isolation for Western blot. On the day of replating, maturation media was supplemented with 10 µM Rock inhibitor (Thermo Fisher Scientific, Waltham, MA, USA) and replaced with fresh maturation media without Rock inhibitor the following day. One third of the media was exchanged twice a week.

### 2.8. Immunofluorescence

Prior to immunostaining, NGN2-derived neurons were fixed with 4% PFA for 20 min at room temperature. Cells were then permeabilized with 0.1% Triton X-100 and blocked for 30 min in blocking solution containing 10% FBS and 1% BSA in DPBS. Following blocking, cells were incubated with primary antibodies (listed in Table 1) diluted in staining solution (0.1% BSA in DPBS) overnight at 4 °C with gentle shaking. The next day, cells were washed three times for five minutes each with staining solution. Alexa Fluor-conjugated secondary antibodies (Table 2) and Hoechst 33342 (Aat Bioquest, Pleasanton, CA, USA) were diluted in staining solution and applied simultaneously for 1 h at room temperature, protected from light and with gentle shaking. After staining, cells were again washed three times for five minutes with staining solution and stored at 4 °C in DPBS until imaging. Imaging was performed using a LSM980 laser scanning confocal microscope (Zeiss, Oberkochen, Germany).

### 2.9. Cell Lysis and Western Blot Analysis

For preparation of cell lysates, cells were washed twice with DBPS on ice. To prevent cell detachment, 50 µL of RIPA buffer was added directly to the plate and cells were collected using a cell scraper (Faust Lab Science, Kamen, Germany). The lysates were then transferred to a centrifuge tube and incubated for 30 min on ice. Lysates were then spun down at 12,000 rpm on a bench centrifuge for 20 min and the supernatant was transferred to a new centrifuge tube and stored at −20 °C until used. Protein concentration was estimated using BCA assay immediately before electrophoresis. BCA absorbance at 562 nm was measured using a Biotek Synergy Neo plate reader (Agilent, Santa Clara, CA, USA).

For PI4KIIIα protein level analysis, 8% SDS-polyacrylamide gels (SDS-PAGE) were used, while 12% SDS-PAGE gels were used for tau protein analysis. Protein samples (30 µg) were prepared by dilution in 2× Laemmli buffer, followed by denaturation at 95 °C for 5 min. Samples were then loaded onto the SDS-PAGE gels. The separation gel was prepared using 0.5 M Tris-HCl (pH 8.8), 10% SDS, Rotiphorese^®^ Gel 30 (Carl Roth, Karlsruhe, Germany), 10% APS, and 10% TEMED in H_2_O, and was preceded by a 5% stacking gel of similar composition, except when using Tris-HCl (pH 6.8). SDS-PAGE was performed using the Mini-PROTEAN^®^ 3 system (Bio-Rad, Hercules, CA, USA). Proteins were transferred overnight at 4 °C onto methanol-activated PVDF membranes. The transfer buffer consisted of 25 mM Tris, 192 mM glycine, and 20% methanol (pH 8.3).

Following transfer, membranes were blocked with blocking buffer composed of 5% milk in TBS-T buffer (50 mM Tris, 150 mM NaCl, 0.1% Tween-20) for 1 h at room temperature. Membranes were then incubated overnight at 4 °C with primary antibodies (Table 1) diluted in blocking solution. After three 10 min washes with TBS-T, membranes were incubated with peroxidase-conjugated secondary antibodies (Table 2) diluted in blocking buffer. Excess secondary antibody was removed with three additional 10 min washes with TBS-T. Protein detection was performed using an ECL chemiluminescence detection reagent (GE Healthcare, Chicago, IL, USA), and signals were captured using the ImageQuant 800 imaging system (GE Healthcare, Chicago, IL, USA). Acquired images were analyzed using ImageJ software.

### 2.10. Tau Internalization Assays (pHrodo-FL-Tau)

After doxycycline induction, neurons were plated on PLO-Laminin 521-coated 96-well multiwell plates at a density of 50,000 cells/well. After one week in maturation media (Neurobasal medium with 1× B27 (Gibco, Thermo Fisher Scientific, Waltham, MA, USA), 2 mM l-glutamine (Gibco, Thermo Fisher Scientific, Waltham, MA, USA), 1 µg/mL laminin (Biolamina, Sundbyberg, Sweden), 20 ng/mL BDNF (Peptrotech, Thermo Fisher Scientific, Waltham, MA, USA), 10 ng/mL GDNF (Peprotech, Thermo Fisher Scientific, Waltham, MA, USA)), *MAPT*-Mut NGN2-derived neurons were treated with the indicated compound concentrations or DMSO vehicle. Compound and DMSO were diluted in maturation media prepared with Neurobasal^TM^ medium minus phenol red (Thermo Fisher Scientific, Waltham, MA, USA), suitable for live imaging. All conditions contained the same final DMSO volume, matched to that of the highest compound concentration by topping up with extra DMSO where needed. Six hours after treatment,10 μg/mL pHrodo-FL-tau seeds were added directly to the culture media containing the treatments.

Twenty-four hours after pHrodo-FL-tau seeds treatment, cells were prepared for live imaging as follows: media was exchanged to maturation media minus phenol red containing LysoTracker^TM^ Deep Red(Thermo Fisher Scientific, Waltham, MA, USA) at a final working concentration of 100 nM for 30 min in total; 20 min after LysoTracker^TM^ incubation, 2 μg/mL Hoechst 33342 was added directly to the media and allowed to incubate with both dyes run for the remaining 10 min. After incubation, cells were washed thrice with Neurobasal^TM^ minus phenol red, and live imaging acquisition was performed immediately after in maturation media without phenol red.

High-content imaging was performed in collaboration with the Technology Development Studio (TDS) at the MPI-CBG (Dresden) by automated image acquisition to avoid observation bias using a Cell Voyager CV7000 system (Yokogawa, Tokyo, Japan) with a 60× objective. Each experimental condition was tested in three technical replicates, with nine images acquired per well, resulting in 27 images per condition per biological replicate. Maximum intensity projections were generated, and pHrodo-FL-tau and LysoTracker™ puncta were quantified using ImageJ software. To assess the uptake of pHrodo-FL-tau seeds, LysoTracker™-positive puncta were segmented, and pHrodo-FL-tau fluorescence intensity was measured within these regions. Fold change was calculated by dividing each test value by the average value of the DMSO control. For statistical significance calculations, all data points were pooled across independent experiments. Samples were tested for normality to choose the appropriate statistical test, which is specified in the figure legend of each graph.

### 2.11. Seeding Assays in NGN2-Induced Neurons

After doxycycline induction, neurons were plated on PLO-Laminin 521-coated 96-well multiwell plates at a density of 50,000 cells/well. Cells were maintained in maturation media for one month, to allow 4R tau isoforms expression. We treated the cells with GSK-F1 or GSK-A1, and we used an equivalent DMSO volume to the compound concentration tested as vehicle control. We also included different untreated controls, including *MAPT*-WT unseeded, *MAPT*-WT seeded, *MAPT*-Mut unseeded and *MAPT*-Mut seeded. For these seeding experiments, we used K18(P301L) seeds at a final concentration of 1 μM. Compound or vehicle treatment was performed during 6 or 24 h, prior to the addition of the K18(P301L) seeds. Tau seeds were directly added to the maturation media containing the compound or vehicle, and media was exchanged 24 h after addition of the seed to fresh maturation media. Every tested condition was tested in three technical replicates.

Seeding in neurons was measured using the HTRF tau aggregation kit (Revvity, Waltham, MA, USA, cat. number 6FTAUPEG). Lysis for HTRF was performed 10 days after addition of K18(P301L) seeds according to manufacturer’s instructions. Briefly, maturation media was carefully removed to prevent cell detachment and 50 μL lysis buffer supplemented with the supplied blocking reagent were added to each well. Cells were mechanically lysed using a multichannel pipette, and the multiwell plate was then placed for one hour at room temperature on an orbital shaker. The plate was then sealed with Parafilm and stored at 4 °C until the following day. The antibodies were diluted according to the kit’s instructions, and 10 μL of the antibody mix (anti-total tau d2 and anti-total tau Eu^3+^-cryptate) was added to 10 μL of cell lysate in a 384-well plate. Each tested well was measured in technical duplicates during HTRF, yielding a total of 6 data points for each independent experiment. We also included a diluent control without antibodies, that was used for background subtraction, and a cryptate control containing only the anti-human tau-Tb^3+^ to check signal at 620 nm. Finally, a negative control using diluent as a sample and both antibodies was used to calculate fold change of 620 nm/665 nm signal ratios.

Fluorescence signal was measured using a Biotek Synergy Neo plate reader (Agilent, Santa Clara, CA, USA), with special filter cubes that allowed simultaneous measurement of the fluorescence at 620 nm and 665 nm. To analyze seeding activity of K18(P301L) between *MAPT*-WT and *MAPT*-Mut neurons, individual values of 620 nm/665 nm ratios of three independent experiments were pooled together and a fold change was calculated by dividing each test value by the average value of the negative assay control. To compare the effect between compound and vehicle, a second fold change was calculated by dividing each test value by the average value of the DMSO control.

## 3. Results

### 3.1. PI4KIII as a Novel Target for Inhibiting the Seeding of Tau Pathology

**Figure 1:**
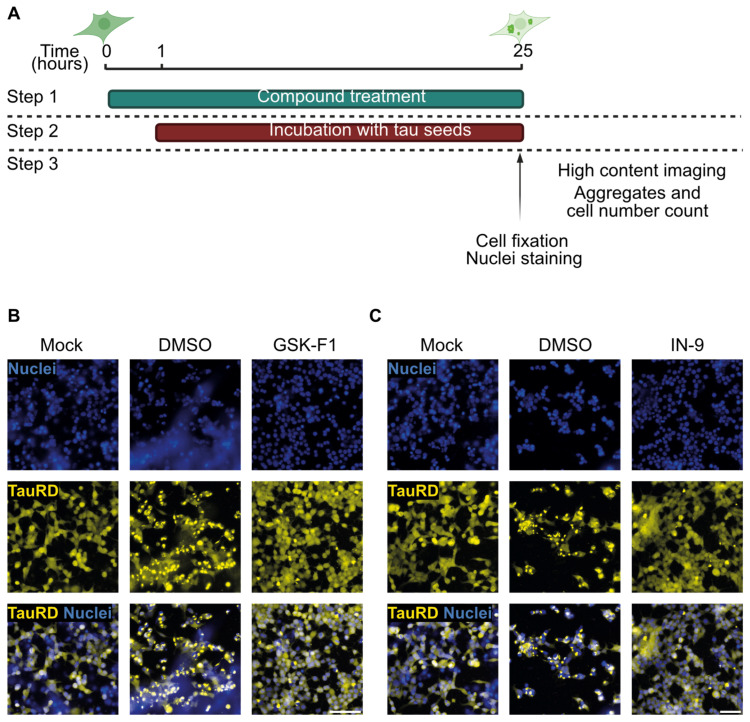
Screening of small molecules on tau biosensor cells. (**A**) Diagram representing the experimental design of the screening campaign. Created in BioRender. Sterneckert, J. (2026) https://BioRender.com/fc5i0r3 (accessed on 8 June 2026). (**B**) Representative images of tau biosensor cells treated with GSK-F1 at 10 μM showing reduction in the number of aggregates relative to the DMSO control. Scale bar = 75 µm. (**C**) Representative images of tau biosensor cells treated with IN-9 at 10 μM showing reduction in the number of aggregates relative to the DMSO control. Since IN-9 was diluted from a 10 mM stock solution in DMSO, the DMSO levels in the 10 µM IN-9 condition was 0.1% of the total volume. For this reason, the DMSO level in the DMSO control well was also 0.1% of the total volume. Scale bar = 50 µm.

**Figure 2:**
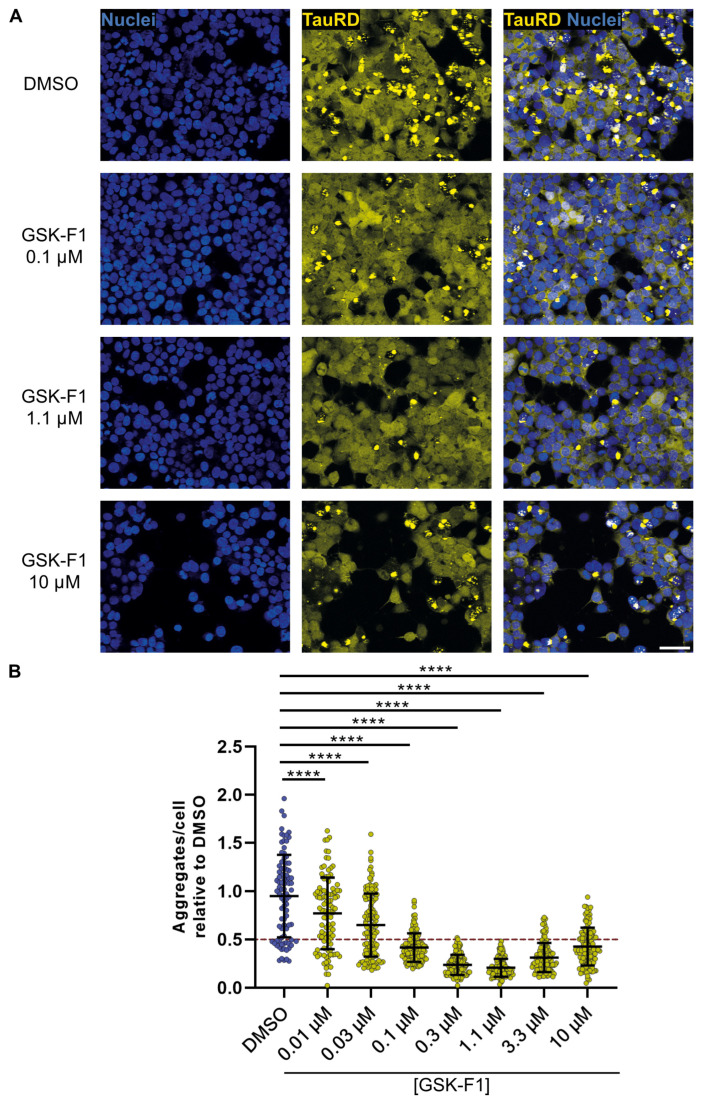
GSK-F1 is the primary hit compound and effectively reduces tau aggregation in biosensor cells. (**A**) Representative fluorescence images of tau biosensor cells treated with the indicated concentration of the PI4KIIIα inhibitor GSK-F1. Scale bar = 50 μm. (**B**) Graph shows mean of all data points of five independent experiments (*N* = 4; 30 images per replicate). Dashed line indicates 50% reduction compared with DMSO-treated controls. Error bars indicate standard deviation. **** indicates *p* < 0.0001 according to two-way ANOVA followed by Dunnett’s multiple comparisons test against the DMSO control. Since GSK-F1 was diluted from a 10 mM stock solution in DMSO, the DMSO levels in the 10 µM GSK-F1 condition was 0.1% of the total volume. For this reason, the DMSO level in the DMSO control well was also 0.1% of the total volume. See Supplementary Information for replicate-specific data as well as ANOVA results.

The TauRD P301S tau biosensor assay represents an in vitro model of tau spreading, in which exogenous seeds induce the formation of intracellular solid aggregates [4]. We used K18 tau fibrils to induce aggregation in tau biosensor cells and test the hypothesis that inhibition of PI4KIII would reduce the seed-induced formation of intracellular tau aggregates. To enhance the efficiency of seeding, we used lipofectamine to transfect tau seeds into biosensor cells. We found that two PI4KIII inhibitors, GSK-F1 and Inhibitor-9 (IN-9) reduced tau aggregation by about 90% relative to the control (Figure 1). A dose–response curve demonstrated that GSK-F1 reduced tau aggregation in a dose-dependent manner with concentrations 0.3–10 µM achieving a reduction greater than 50% in aggregate formation (Figure 2). A concentration of >10 μM GSK-F1 was associated with reduced cell number, indicating cytotoxicity. These data suggest that inhibition of PI4KIIIα could efficiently reduce tau seeded aggregation in tau biosensor cells at nanomolar concentrations with 10 µM being the maximally tolerated dose in this study.

**Figure 3:**
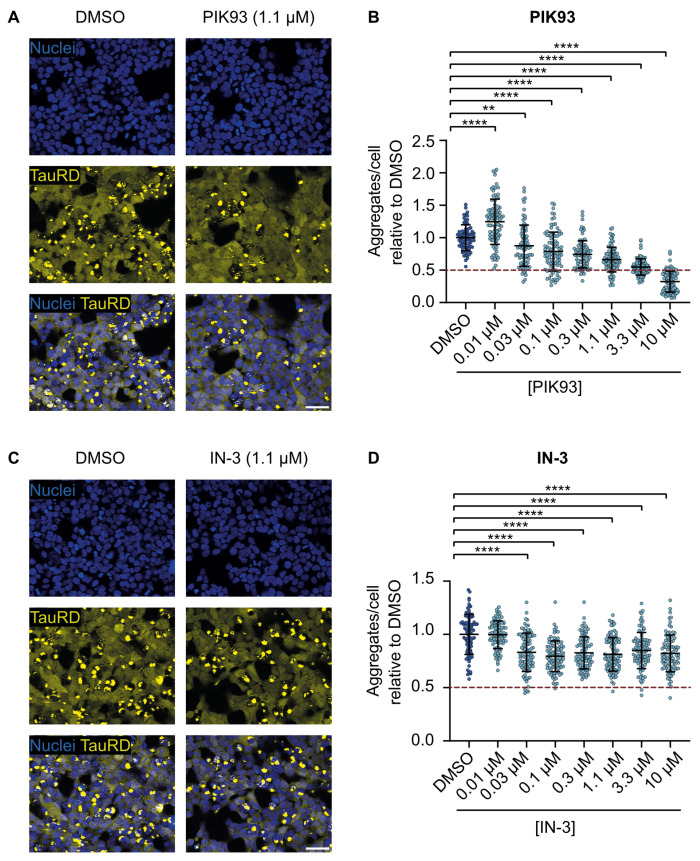
Effects of PI4KIII inhibitors PIK93 and IN-3 on tau aggregation in tau biosensor cells. (**A**) Representative fluorescence images of tau biosensor cells treated with the indicated concentration of the PI4KIIIβ inhibitor PIK93. (**B**) Graph shows quantification of tau aggregates divided by the total number of nuclei; all independent data points of three independent experiments (*N* = 3; 30 images per replicate) are displayed. ****, ** indicate *p* < 0.0001, *p* < 0.01, respectively, according to two-way ANOVA followed by Dunnett’s multiple comparisons test against the DMSO control. Error bars represent standard deviation. (**C**) Representative fluorescence images of tau biosensor cells treated with the indicated concentration of the PI4KIIIβ inhibitor IN-3. (**D**) Graph shows quantification of tau aggregates divided by the total number of cells; all data points of four independent experiments (*N* = 3; 30 images per replicate) are displayed. Dashed line indicates 50% reduction compared with DMSO-treated controls. **** indicate *p* < 0.0001 according to two-way ANOVA followed by Dunnett’s multiple comparisons test against the DMSO control. Error bars represent standard deviation. Scale bar = 50 μm. Since PIK93 and IN-3 were diluted from a 10 mM stock solution in DMSO, the DMSO levels in the 10 µM condition was 0.1% of the total volume. For this reason, the DMSO level in the DMSO control well was also 0.1% of the total volume. See Supplementary Information for replicate-specific data as well as ANOVA results.

To gain insights on the relative contribution of PI4KIIIα and PI4KIIIβ in tau aggregation, we used the tau biosensor cell assay to test four compounds with different levels of activity against PI4KIIIα and PI4KIIIβ (Table 3). PIK93 reduced tau aggregation by more than 50% in comparison to the DMSO control only at the highest concentration tested (Figure 3). Since biochemical data shows that PIK93 inhibits PI4KIIIα at concentrations above 1 µM (Table 3), this data suggests that PI4KIIIα may be mediating the reduction in tau aggregation. Consistent with this idea, IN-3 (Table 3), which has not been shown to have any biochemical effect on PI4KIIIα activity, failed to reduce tau seeding by more than 20% at all tested concentrations (Figure 3C,D). Together, these results suggest that inhibition of PI4KIIIα may be essential for seed-induced aggregation in tau biosensor cells.

**Figure 4:**
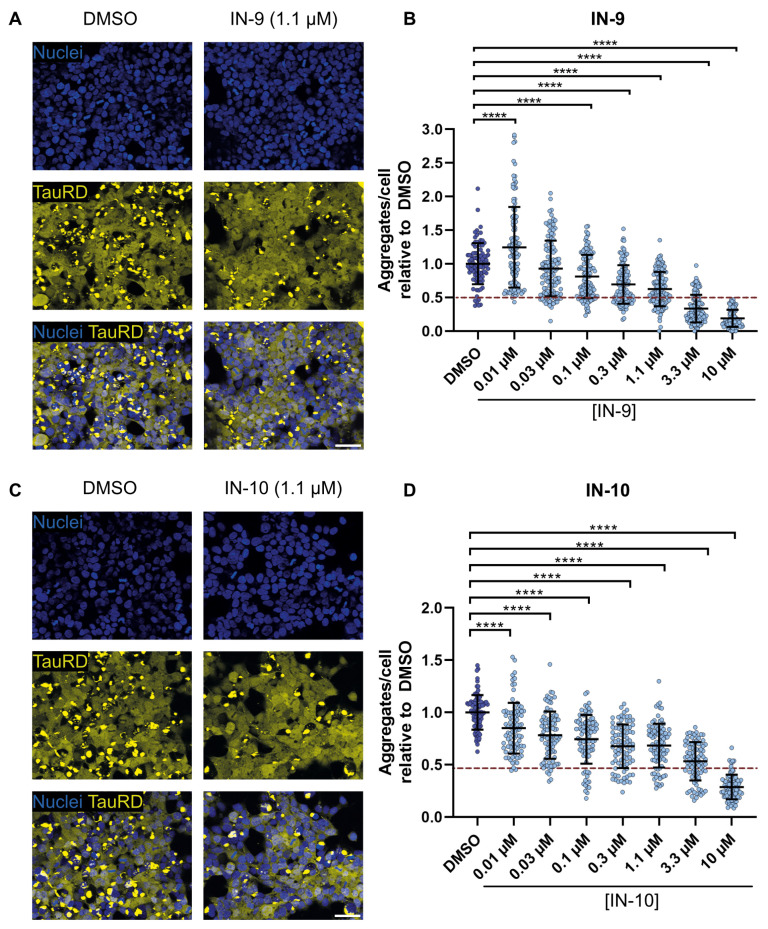
IN-9 and IN-10 effectively reduce tau aggregation in tau biosensor cells. (**A**) Representative fluorescence images of tau biosensor cells treated with the indicated concentration of IN-9. (**B**) Graph shows quantification of tau aggregates divided by the total number of nuclei; all independent data points of five independent experiments (*N* = 4; 30 images per replicate) are displayed. Dashed line indicates 50% reduction compared with DMSO-treated controls. **** indicates *p* < 0.0001. Error bars represent standard deviation. (**C**) Representative fluorescence images of tau biosensor cells treated with the indicated concentration of the PI4KIIIβ inhibitor IN-10. (**D**) Graph shows quantification of tau aggregates divided by the total number of tau biosensor cells; all data points of four independent experiments (*N* = 4; 30 images per replicate) are displayed. Dashed line indicates 50% reduction compared with DMSO-treated controls. **** indicate *p* < 0.0001. Error bars represent standard deviation. For all experiments statistical significance according to two-way ANOVA followed by Dunnett’s multiple comparisons test against the DMSO control. Images scale bar = 50 μm. Since IN-9 and IN-10 were diluted from 10 mM stock solutions in DMSO, the DMSO levels in the 10 µM condition was 0.1% of the total volume. For this reason, the DMSO level in the DMSO control well was also 0.1% of the total volume. See Supplementary Information for replicate-specific data as well as ANOVA results.

To further confirm this hypothesis, we tested dose response curves for IN-9 and IN-10, which inhibit PI4KIIIβ at concentration lower than 10 nM, yet inhibit PI4KIIIα at concentrations higher than 1 µM (Table 3). Consistent with our hypothesis, we found that IN-9 and IN-10 reduced tau aggregation in biosensor cells only when used at concentrations higher than 1 µM (Figure 4). Therefore, taken together, the data from these 5 different small molecules suggest that inhibition of PI4KIIIα, rather than PI4KIIIβ, reduces the seeding of tau aggregation in biosensor cells.

### 3.2. Knock-Down of PI4KIIIα Reduces Tau Seeding Similar to Small Molecule Inhibition

**Figure 5:**
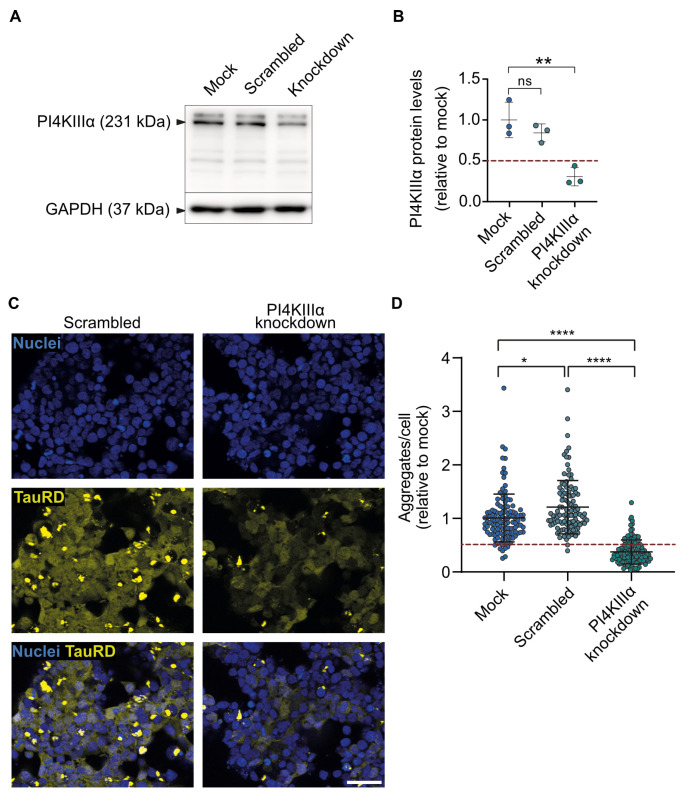
PI4KIIIα validation as target to inhibit tau seeded aggregation. (**A**) Representative immunoblot of PI4KIIIα and GAPDH protein expression in tau biosensor cells transfected with empty liposomes (mock), scrambled or siRNA, 48 h after transfection. Original uncropped blot is shown in Supplementary Figure S11. (**B**) Quantification of relative PI4KIIIα expression of scrambled- or siRNA-treated cells compared to mock condition. PI4KIIIα protein levels were normalized with GAPDH. Data points of three independent knock-down experiments (*N* = 3; 30–40 images per replicate). ** indicates *p* < 0.01. ns indicates not significant. Error bars represent standard deviation. (**C**) Representative fluorescence images of tau biosensor cells seeded with K18 fibrils after 48 h treatment with scrambled or siRNA. Scale bar = 50 µm. (**D**) Quantification of tau aggregates divided by the total number of cells of tau biosensor cells transfected with empty liposomes (mock), scrambled and siRNA. Dashed line indicates 50% reduction compared with DMSO-treated controls. **** indicate *p* < 0.0001 and * indicate *p* < 0.05. Error bars represent standard deviation. For all experiments statistical significance according to two-way ANOVA followed by Dunnett’s multiple comparisons test. See Supplementary Information for replicate-specific data as well as ANOVA results.

Screening small molecules on tau biosensor cells revealed that PI4KIIIα might play a role in seed-induced tau aggregation. To further test this hypothesis, we performed a knock-down of PI4KIIIα and characterized the effect on tau aggregation in the tau biosensor assay. We optimized PI4KIIIα knock-down using small interference RNA (siRNA) and a scrambled control. We transfected tau biosensor cells with either scrambled and siRNA against PI4KIIIα and lysed after 48 h. Western blot analysis showed that siRNA significantly reduced PI4KIIIα protein levels by about 75% (Figure 5A,B).

Next, we tested the effects of PI4KIIIα knock-down on tau seeding using tau biosensor cells. To do this, we first transfected the siRNA or the scrambled control and waited 48 h until transfecting tau pathogenic seeds. Aggregates formation was measured 24 h after the addition of the tau seeds. We observed that knocking down PI4KIIIα protein was associated with a more than 50% reduction in aggregates formation in tau biosensor cells when compared with the conditions transfected with empty liposomes (mock) or with the scrambled sequence (Figure 5). Taken together with the small molecule data, these results indicate that PI4KIIIα is essential for seed-induced tau aggregation in biosensor cells.

### 3.3. PI4KIIIα Inhibition Reduces the Seeding Activity of Full-Length Tau Fibrils

**Figure 6:**
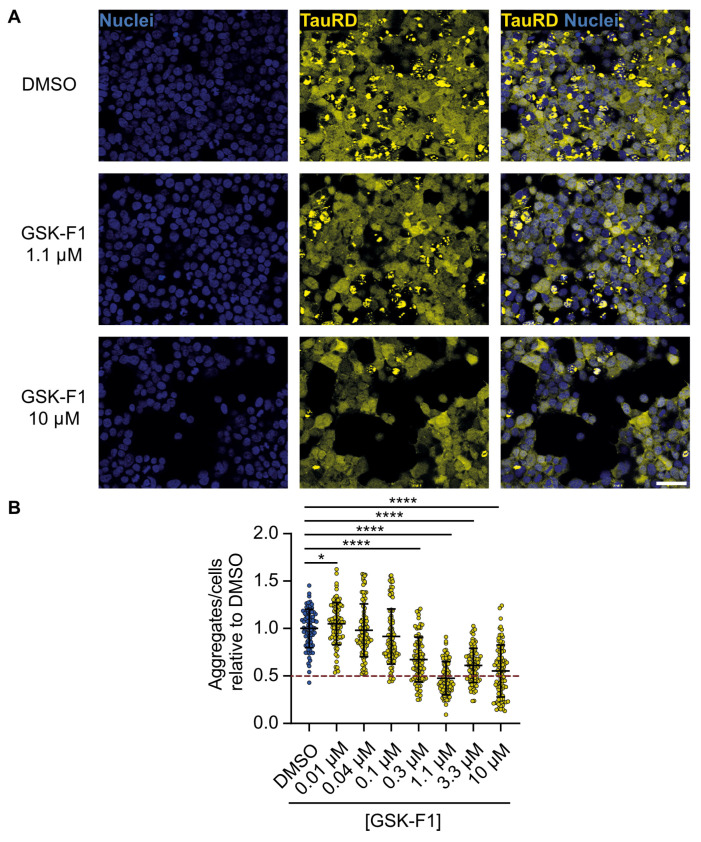
GSK-F1 effectively reduces tau aggregation in biosensor cells seeded with FL-tau fibrils. (**A**) Representative fluorescence images of tau biosensor cells treated with the indicated concentration of the PI4KIIIα inhibitor GSK-F1. Scale bar = 50 μm. (**B**) Graph shows mean of all data points of three independent experiments (*N* = 3; 30 images per replicate). Error bars indicate standard deviation. Dashed line indicates 50% reduction compared with DMSO-treated controls. **** and * indicate *p* < 0.0001 and *p* < 0.05, respectively, according to two-way ANOVA followed by Dunnett’s multiple comparisons test against the DMSO control. Since GSK-F1 was diluted from a 10 mM stock solution in DMSO, the DMSO levels in the 10 µM GSK-F1 condition was 0.1% of the total volume. For this reason, the DMSO level in the DMSO control well was also 0.1% of the total volume. See Supplementary Information for replicate-specific data as well as ANOVA results.

Previous experiments utilized K18 tau fibrils to induce aggregation in tau biosensor cells. While K18 fibrils are a useful tool, they represent an artificial system, as they comprise only a truncated fragment of tau protein. We hypothesized that full-length 2N4R tau (FL-tau) would also show PI4KIIIα-dependent seeding in the biosensor cell assay. To test this idea, we adapted the fibrillation protocol to produce FL-tau fibrils, which were sonicated to generate seeds that induced aggregation in tau biosensor cells (Supplementary Figure S1). The PI4KIIIα inhibitor GSK-F1 inhibited FL-tau seed-induced aggregation in tau biosensor cells in a dose-dependent manner (Figure 6), but the magnitude of the effect was reduced compared with K18-derived seeds.

### 3.4. PI4KIIIα Inhibition Reduces the Levels of Intracellular FL-Tau Seeds

PI4KIIIα is responsible for maintaining PI(4)P levels at the plasma membrane [20], and PI(4)P at plasma membrane serve as precursors for PI(4,5)P_2_ synthesis, which plays several important roles in endocytosis [9,21,22]. For these reasons, we hypothesized that inhibition of PI4KIIIα might prevent the seed-induced tau aggregation by reducing internalization of tau seeds.

Next, we aimed to directly visualize intracellular FL-tau seeds after being added exogenously. In a preliminary experiment, we found that tau aggregation within biosensor cells as first detected between 5 and 7 h after adding FL-tau, which is why we chose 6 h as the experimental time point (Supplementary Figure S2). To assess intracellular FL-tau seeds, we fluorescently labeled tau fibrils by covalently binding a fluorophore obtaining fluorescently labeled FL-tau (FL-tau-488) and imaged HEK293T cells, which are the parent cell line for tau biosensor cells, six hours after transfection. Wheat germ agglutinin (WGA) was used to visualize the cellular membranes. Confocal imaging showed that GSK-F1 induced a statistically significant reduced amount of tau puncta per cell compared to vehicle alone (Supplementary Figure S3), suggesting that PI4KIIIα inhibition reduces the levels of tau-FL-488 fibrils inside HEK293T cells at 6 h. Although this result is preliminary since it was only performed twice, the results could explain the reduced seeding activity observed in the aggregation assays in tau biosensor cells (Figure 1).

### 3.5. A Human iPS Cell-Derived Cortical Neuron Model of Seed-Induced Formation of Pathological Tau Assemblies

To complement and further validate our results using tau biosensor cells, we sought to generate a model of seed-induced formation of pathological tau assemblies using iPS cell-derived cortical neurons in order to test the efficacy of PI4KIIIα inhibition. To do this, we used an iPS cell line (hereafter referred to as *MAPT*-Mut) with the IVS10+16 mutation, which increases the levels of the 4R splice variant, as well as the P301S mutation, which increases tau aggregation propensity [16]. An isogenic iPS cell line lacking these mutations was used as a control (hereafter referred to as *MAPT*-WT) [16]. To facilitate efficient cortical neuron differentiation, an inducible NGN2 expression cassette was knocked into the *AAVS1* safe harbor locus (Supplementary Figure S4). Mutant iPS cells were homozygous for the iNGN2 cassette while wild type (WT) controls were heterozygous (Supplementary Figure S5). Metaphase spread analysis confirmed a euploid karyotype (Supplementary Figure S5D). To quantify neuronal differentiation efficiency, we analyzed the number of cells that expressed the telencephalic marker FOXG1 together with the neuronal marker tubulin beta 3 (TUBB3). The results show that *MAPT*-Mut neurons manifested a 98% differentiation efficiency, whereas *MAPT*-WT neurons showed a higher variability with an average of 82% of the cells expressing both markers (Supplementary Figure S6). The higher differentiation efficiency variability of the *MAPT*-WT cell line could be explained by the presence of one unedited *AAVS1* allele (Supplementary Figure S6).

Next, we analyzed the expression of 4R tau isoforms at different days of maturation. We collected cell lysates at maturation day 7, 14, and 30 and analyzed 4R tau protein levels by Western blot. The results showed that *MAPT*-Mut neurons had a higher expression of 4R tau at all the time points that were tested (Supplementary Figure S7A,B). Furthermore, we also visualized 4R tau expression in neurons by performing an immunostaining using the same 4R tau in *MAPT*-WT and *MAPT*-Mut neurons at maturation day 30. The results show significantly increased expression of 4R tau in *MAPT*-Mut neurons at this time point of maturation (Supplementary Figure S7C,D). It is possible that the difference in the differentiation efficiency between *MAPT*-WT and *MAPT*-Mut contributes to the observed difference in 4R tau expression levels. Nevertheless, these results dare demonstrate that the *MAPT*-Mut iPS cell-derived cortical neurons are suitable for establishing an assay for seeding of pathological tau assemblies.

### 3.6. PI4KIIIα Inhibition Reduces the Levels of Exogenous Tau Seeds Within iPS Cell-Derived Cortical Neurons

**Figure 7:**
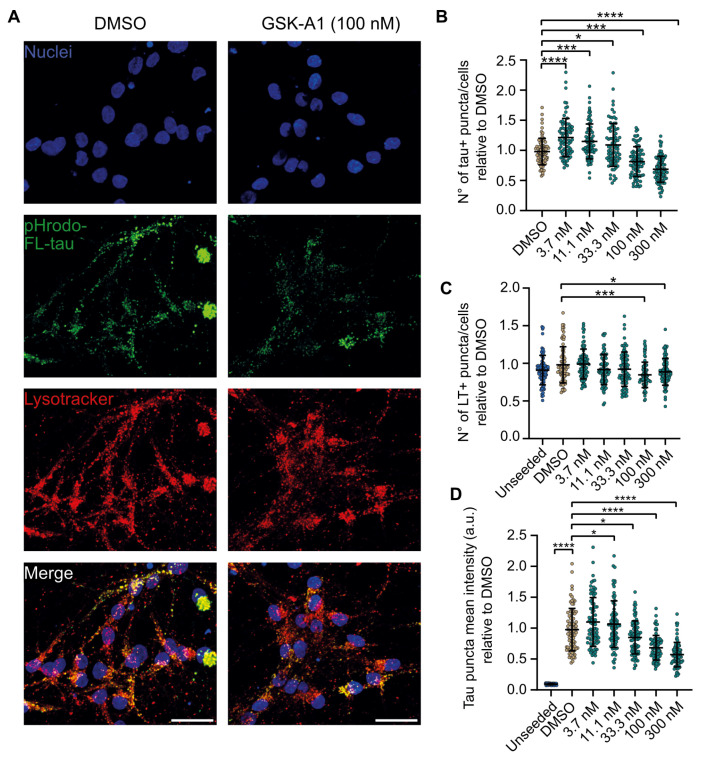
PI4KIIIα inhibition with GSK-A1 reduces intracellular levels of pHrodo-FL-tau seeds after being exogenously added to iPS cell-derived neurons. (**A**) Diagram shows the experimental design to study intracellular levels of pHrodo-FL-tau seeds in one week old MAPT-Mut neurons. Created in BioRender. Sterneckert, J. (2026) https://BioRender.com/kuozzcr (accessed on 8 June 2026). (**B**) Representative fluorescence images of MAPT-Mut neurons on maturation day 7 treated with the indicated concentration of GSK-A1 or the DMSO control and pHrodo-FL-tau seeds. Scale bar = 50 μm. (**C**,**D**) Graphs showing the mean of all data points of three independent experiments (*N* = 3; 27 images per replicate). Error bars indicate standard deviation. ****, *** and * indicate *p* < 0.0001, *p* < 0.001 and *p* < 0.05, respectively, according to two-way ANOVA followed by Dunnett’s multiple comparisons test against the DMSO control. Since GSK-A1 was diluted from a 10 mM stock solution in DMSO, the DMSO levels in the 300 nM condition was 0.003% of the total volume. For this reason, the DMSO level in the DMSO control well was also 0.003% of the total volume. See Supplementary Information for replicate-specific data as well as ANOVA results.

Next, we characterized the effects of PI4KIIIα inhibition on the intracellular levels of FL-tau seeds after being exogenously added to iPS cell-derived cortical neurons. Since transfection using lipofectamine represents an artificial internalization system, exogenous seeds were added to iPS cell-derived cortical neurons in the absence of any transfection reagents. iPS cell-derived cortical neurons were treated for 6 h with either GSK-F1 at 1 μM and GSK-A1 at 0.1 μM, which was well tolerated (Supplementary Figure S8). To facilitate visualization of intracellular seed levels, FL-tau seeds were labeled with pHrodo, which is a pH-sensitive fluorescent indicator, enabling visualization of seeds within endosomes and lysosomes, and added to iPS cell-cortical neurons for 24 h (Figure 7A). Confocal microscopy was used to quantify the pHrodo-FL-tau positive puncta and lysotracker-positive acidic endosomes/lysosomes normalized against the total cell number.

GSK-A1 showed a concentration dependent decrease in the number of intracellular levels of pHrodo-FL-tau seeds compared with DMSO, and GSK-F1 showed lower levels when tested at 3 µM (Figure 7 and Supplementary Figure S9). The number of lysotracker positive puncta was not altered, suggesting that late endosomes and lysosomes remained intact (Figure 7C and Supplementary Figure S9C). Interestingly, the pHrodo-FL-tau seeds intensity inside lysotracker positive puncta showed a significant dose-dependent effect, suggesting that PI4KIIIα inhibition may decrease the amount of tau seeds in acidic compartments (Figure 7D and Supplementary Figure S9D).

These results suggest that PI4KIIIα inhibition can efficiently reduce the amount of intracellular FL-tau seed after being exogenously added to iPS cell-derived cortical neurons. Since pHrodo is pH-dependent, we cannot rule out the possibility that PI4KIIIα modifies the degradation of internalized tau seeds. Importantly, the compound effect correlates with the potency of inhibiting PI4KIIIα. GSK-A1 is more potent than GSK-F1 against PI4KIIIα, and, similarly, GSK-A1 induced a more pronounced reduction in the intracellular levels of tau seeds in iPS cell-derived cortical neurons than GSK-F1.

### 3.7. Exogenous Seeds Induce Tau Pathology in iPS Cell-Derived Cortical Neurons

**Figure 8:**
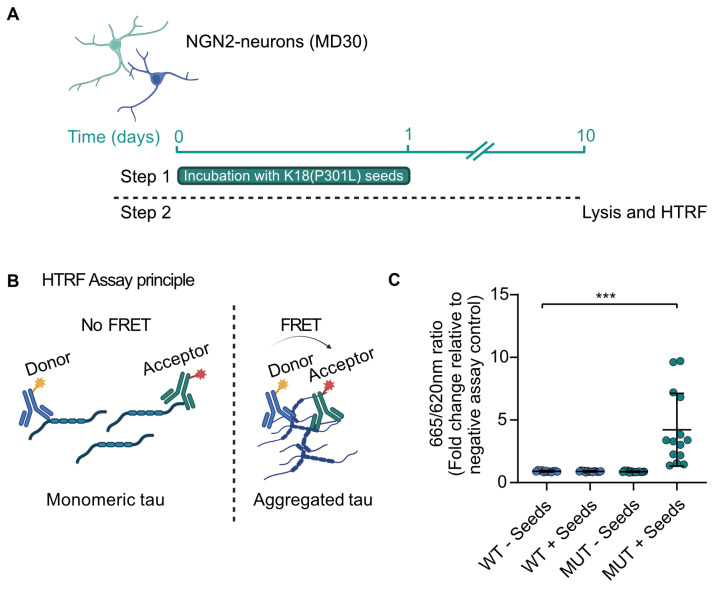
HTRF assay detects endogenous tau assemblies in iPS cell-derived neurons. (**A**) Diagram illustrating the experimental design of the tau assembly assay, in which neurons were treated for 24 h with K18(P301L)-tau seeds and lysis for HTRF was performed 10 days after addition of the seeds. Created in BioRender. Sterneckert, J. (2026) https://BioRender.com/c631363 (accessed on 8 June 2026). (**B**) Scheme of the HTRF tau aggregation assay principle, which shows no FRET signal when tau is not aggregated, and a positive FRET signal when donor and acceptor antibodies are in close proximity due to tau aggregation. Created in BioRender. Sterneckert, J. (2026) https://BioRender.com/nisy3ag (accessed on 8 June 2026). (**C**) Tau aggregation HTRF in maturation day 30 MAPT-WT and MAPT-Mut neurons seeded or unseeded with K18(P301L)-tau seeds. Graph shows mean of all data points of three independent experiments (*N* = 3; 4–6 data points per replicate). Error bars represent standard deviation. *** indicates *p* < 0.001 according to two-way ANOVA followed by Dunnett’s multiple comparisons test. See Supplementary Information for replicate-specific data as well as ANOVA results.

Subsequently, we sought to generate a model in which exposure of cortical neurons to exogenous tau seeds induced aggregation of endogenous tau protein. To do this, we utilized homogeneous time-resolved fluorescence (HTRF), which enables the detection of a fluorescent FRET signal only in the presence of endogenous tau assemblies (Figure 8A,B) with the signal intensity proportional to the amount of tau aggregates present. The antibodies provided in the kit recognize epitopes on the tau protein outside the microtubule-binding domain, specifically within the proline-rich region. To avoid detection by the HTRF antibodies, we used tau seeds generated from mutant K18(P301L)-tau seeds, which were previously shown by another research team to possess a higher seeding potential than wild-type K18-tau seeds [23]. This approach allowed us to initiate aggregation without the seeds being recognized by the HTRF detection kit. As a result, the signal detected by the HTRF assay reflected only the intracellular tau assemblies formed by endogenously expressed tau protein in iPS cell-derived neurons.

To establish the seeding assay, we added K18(P301L)-tau seeds for 24 h to *MAPT*-WT and *MAPT*-Mut neurons at day 30 of maturation. Ten days after adding the K18(P301L)-tau seeds, cells were lysed according to manufacturer’s instructions (Figure 8A). After lysis, HTRF was measured and we observed a significantly increased HTRF signal in *MAPT*-Mut seeded neurons in comparison to the unseeded *MAPT*-WT control neurons. Adding tau seeds to *MAPT*-WT did not show any increase in the HTRF signal, demonstrating that the kit does not recognize the exogenous seeds and, more importantly, that seeding only triggered aggregation in *MAPT*-Mut neurons expressing 4R tau isoforms (Figure 8C). These results suggest that we were able to detect endogenous tau assemblies in iPS cell-derived mutant cortical neurons ten days after exposing them to mutant K18 tau seeds. It should be noted that further experiments would be needed to assess if the HTRF signal corresponds to tau aggregates or, for example, to tau oligomers.

### 3.8. PI4KIIIα Inhibitor GSK-A1 Reduces Induction of Tau Pathology in Human Cortical Neurons

Our next aim was to test the hypothesis that PI4KIIIα inhibition would reduce tau seeding activity in iPS cell-derived cortical neurons. For this reason, 30 days mature *MAPT*-Mut neurons were treated with 1 μM GSK-F1 or 0.1 μM GSK-A1, and their respective DMSO controls, for a total time of six hours before addition of K18(P301L)-tau seeds. Seeds were removed from the media 24 h after being added to the media and endogenous tau assemblies were measured ten days after tau seeds addition to the neuronal maturation media (Supplementary Figure S10). From the results, we can observe that the treatment with GSK-F1 and GSK-A1 did not reduce significantly in the tau assembly under these conditions (Supplementary Figure S10).

**Figure 9:**
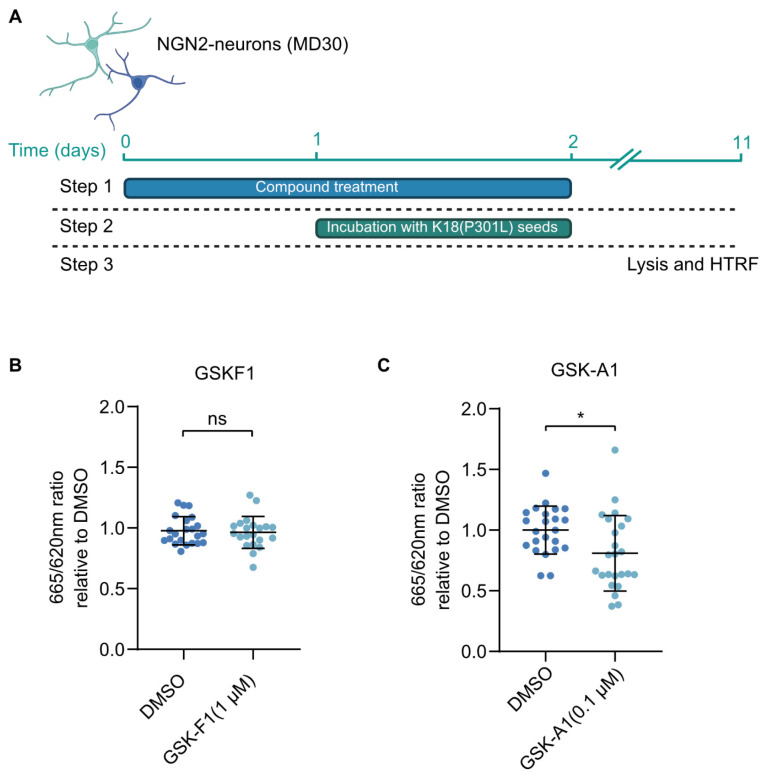
24 h treatment with PI4KIIIα inhibitor GSK-A1 reduces tau assembly formation in MAPT-Mut neurons. (**A**) Diagram illustrating the experimental design of the tau assembly assay, in which neurons were treated for 24 h with K18(P301L)-tau seeds and lysis for HTRF was performed 10 days after addition of the seeds. Compounds were added 24 h prior to the addition of the seeds and were present in the media during the total duration of the treatment with K18(P301L)-tau seeds. Tau aggregation HTRF in 30 days mature MAPT-Mut neurons treated with the indicated concentrations of (**B**) GSK-F1 or (**C**) GSK-A1. Diagram created in BioRender. Sterneckert, J. (2026) https://BioRender.com/i1lyage (accessed on 8 June 2026). Graphs show the mean of all data points of three independent experiments (*N* = 4; 6 data points per replicate). Error bars represent standard deviation. * indicates *p* < 0.05 according to two-way ANOVA. ns indicates not significant. Since GSK-F1 and GSK-A1 were diluted from 10 mM stocks in DMSO, the DMSO levels in the 1 µM and 0.1 µM conditions were 0.01% and 0.001%, respectively, of the total volume. For this reason, the DMSO level in the DMSO control well was also 0.01% and 0.001% of the total volume for GSK-F1 and GSK-A1, respectively. See Supplementary Information for replicate-specific data as well as ANOVA results.

Since the timing of tau assembly formation is different between tau biosensor cells and iPS cell-derived cortical neurons, we hypothesized that the duration of compound treatment needed to be extended to observe the effect of PI4KIIIα inhibition. Therefore, we performed treatment of iPS cell-derived cortical neurons with GSK-F1 and GSK-A1 for 24 h prior to the addition of the seeds (Figure 9A). When measuring tau assembly formation using the HTRF assay ten days after the first contact with the seeds, we observed that the treatment with GSK-F1 did not alter endogenous tau assembly formation (Figure 9B). In contrast, the treatment with GSK-A1, which can more potently target PI4KIIIα, showed a statistically significant reduction in endogenous tau assembly (Figure 9C). Importantly, these results are consistent with the results obtained in the pHrodo-FL-tau seed assays. A reduction in the intracellular levels of tau exogenous seeds is also accompanied by a reduced seeding after treatment with GSK-A1, which in general appears more potent in reducing tau-associated phenotypes in comparison with GSK-F1.

Together, these results suggest that the treatment of iPS cell-derived cortical neurons with GSK-A1 reduces the internal levels of exogenous tau seeds as well as induction of tau assembly formation. These findings suggest that PI4KIIIα is an important regulator of seed-induced formation of pathological tau assemblies and aggregation.

## 4. Discussion

Tauopathies represent a major group of neurodegenerative diseases, affecting millions of people worldwide, and inhibition of tau prion-like spreading represents a one strategy to protect neurons against disease progression. PI4KIIIα was originally identified in siRNA screens as a key factor required for hepatitis C virus entry and replication [24,25], which led to development of PI4KIIIα inhibitors [15], including GSK-F1 and GSK-A1. We demonstrated that pharmacological inhibition as well as siRNA-mediated reduction in PI4KIIIα protein levels effectively reduces the seeding of tau aggregation in tau biosensor cells as well as genetically engineered iPS cell-derived cortical neurons harboring pathogenic *MAPT* mutations.

The spreading of tau pathology can have detrimental effects on tau’s physiological function. Although, tau’s best studied function is its binding to neuronal microtubules, increasing evidence suggest that tau also has nuclear functions, including regulation of ribosomal RNA transcription, chromatin structure, and DNA damage [26,27,28]. Future work could test if inhibition of tau pathological spreading by targeting PI4KIIIα would be neuroprotective and restore these tau functions.

One possible mechanism by which PI4KIIIα inhibitors reduce the seeding of tau pathology is by reducing the internalization of extracellular tau seeds. PI4KIIIα is a key enzyme responsible for generating PI(4)P at the plasma membrane. A potential downstream consequence of reduced PI(4)P availability is a corresponding decrease in PI(4,5)P_2_ levels, which could in turn impair clathrin-mediated endocytosis of tau seeds. It is also important to note that tau seed internalization is rather a complex cellular process. At least two studies have shown that also macropinicytosis and bulk endocytosis are important contributors to tau internalization in neurons [8,29]. Since pathogenic tau assemblies are structurally diverse, different types of seeds may use different mechanisms to enter neurons. However, it is important to note that additional experiments would be needed to test this hypothesis, and additional mechanisms may also play a role, including degradation of internalized seeds and regulation of the abundance of endogenous tau protein.

Our iPS cell-based experiments also have important limitations. First, we relied on the HTRF assay to quantify tau aggregation within iPS cell-derived neurons. However, additional experiments are needed to confirm if this HTRF signal corresponds exclusively to changes in tau aggregation or, alternatively, to changes in formation of soluble tau oligomers or may reflect changes in the abundance of intracellular tau protein levels. In addition, iPS cell-derived neurons are relatively immature, and it is generally challenging to fully recapitulate tau pathology phenotypes, which are associated with mature and aged neurons. Thus, additional experiments are needed to validate our findings, particularly in mature aged neurons.

Developing a therapeutic strategy based on targeting PI4KIIIα would be challenging since multiple studies have shown an association with toxicity. Biallelic loss-of-function mutations in PI4KIIIα are associated with several human diseases [30], including neurological, intestinal, and immunological disease [31,32], suggesting that strong reductions in PI4KIIIα levels or activity are toxic. Consistent with this idea, oral administration of a kinase inhibitor in mice caused epithelial cell degeneration in the gastrointestinal tract, and animals died with what appeared to be cardiovascular collapse [17]. However, to our knowledge, clinical phenotypes are only associated with biallelic mutations, and, in mice, no adverse effects were reported in animals with a heterozygous knockout of *Pi4ka* [17,33]. Thus, it is tempting to speculate that partial reductions in PI4KIIIα could be sufficient to reduce seed-induced tau aggregation without inducing toxicity. Consistent with this idea, we found that nanomolar concentrations of PI4KIIIα inhibitors were sufficient to reduce the seed-induced tau aggregation without inducing toxicity in biosensor cells as well as iPS cell-derived cortical neurons. One strategy to do this could be an allosteric PI4KIIIα inhibitor. For example, PIKfyve is a lipid kinase regulating endosome recycling and knockouts are lethal. In contrast, the allosteric PIKfyve inhibitor apilimod is well tolerated and is neuroprotective in multiple models [34,35,36]. Nevertheless, these ideas remain speculative at this stage.

We conclude that PI4KIIIα plays an important role in regulating the internal levels of exogenous tau seeds as well as the seed-induced formation of pathological tau assemblies and aggregation. There is a clear correlation of PI4KIIIα inhibition and the inhibition of tau aggregation. These findings raise the possibility that PI4KIIIα activity could serve as a therapeutic point of intervention to limit pathological tau propagation, but further work is required to reduce possible on-target toxicity. Still, it needs to be understood from a pharmacological perspective, if PI4KIIIα allosteric or partial inhibition can be safely achieved in the brain, whereas systemic inhibition of PI4KIIIα apparently causes toxicity in rodents.

## 5. Conclusions

PI4KIIIα regulates seed-induced aggregation in biosensor cells as well as seed-induced tau assemblies in human iPS cell-derived cortical neurons. This reduction correlates with decreased levels of internalized tau seeds. Additional experiments are needed to assess if this observation is caused by decreased uptake, increased degradation, or altered processing of tau seeds. Future work is also needed to overcome the toxicity associated with PI4KIIIα inhibition.

## Figures and Tables

**Table 1:** List of primary antibodies used for Western blot (WB) and immunofluorescence (IF).

Antibody	Dilution	Company	Catalog Number
Rabbit anti-PI4KA	1:1000 (WB) 1:200 (IF)	Abcam (Cambridge, UK)	ab111565
Mouse anti-Total Tau	1:1000 (WB) 1:100 (IF)	Thermo Fischer Scientific (Waltham, MA, USA)	MN1000
Rabbit anti-4R Tau	1:1000 (WB)	Abcam (Cambridge, UK)	Ab218314
Rabbit anti-GAPDH	1:10,000 (WB)	Cell signaling Technology (Danvers, MA, USA)	2118S
Rabbit anti-FOXG1	1:300 (IF)	Abcam (Cambridge, UK)	ab18259
Mouse anti-TUBB3	1:1000 (IF)	Biolegend (San Diego, CA, USA)	801202
Chicken anti-Map2	1:2000 (IF)	Abcam (Cambridge, UK)	Ab92434

**Table 2:** List of secondary antibodies.

Antibody	Dilution	Company	Catalog Number
AlexaFluor 488 donkey anti-mouse	1:1000	Thermo Fisher Scientific (Waltham, MA, USA)	A21202
AlexaFluor 568 donkey anti-mouse	1:1000	Thermo Fisher Scientific (Waltham, MA, USA)	A10037
AlexaFluor 647 donkey anti-mouse	1:1000	Thermo Fisher Scientific (Waltham, MA, USA)	A31571
AlexaFluor 488 donkey anti-rabbit	1:1000	Thermo Fisher Scientific (Waltham, MA, USA)	A21206
AlexaFluor 568 donkey anti-rabbit	1:1000	Thermo Fisher Scientific (Waltham, MA, USA)	A10042
AlexaFluor 647 donkey anti-rabbit	1:1000	Thermo Fisher Scientific (Waltham, MA, USA)	A31573
AlexaFluor 488 goat anti-chicken	1:1000	Thermo Fisher Scientific (Waltham, MA, USA)	A11039
AlexaFluor 555 goat anti-chicken	1:1000	Thermo Fisher Scientific (Waltham, MA, USA)	A21437
Peroxidase-conjugated anti-mouse	1:10,000	Jackson ImmunoResearch (West Grove, PA, USA)	715-035-150
Peroxidase-conjugated anti-rabbit	1:10,000	Jackson ImmunoResearch (West Grove, PA, USA)	715-035-152

**Table 3:** PI4KIII inhibitors and their reported in vitro IC_50_. The table shows the differential efficacy of the different inhibitors in specifically blocking the kinase activity of PI4KIIIα and PI4KIIIβ. Inhibitors with different activities against were selected in order to assess their individual contributions to tau seed-induced aggregation. For clarity, IC_50_ below 100 nM are shown in green and values at or above 1 µM are shown in red.

Name	PI4KIIIα IC_50_	PI4KIIIβ IC_50_	Citations
GSK-A1	3.2 nM	63 nM	[17]
GSK-F1	16 nM	1 µM	[17]
PIK93	1.1 µM	19 nM	[18,19]
IN-3	Not described	4.4 nM	[18]
IN-9	2.6 µM	7 nM	[18]
IN-10	3 µM	3.6 nM	[18]

## Data Availability

The original contributions presented in this study are included in the article/Supplementary Material. Further inquiries can be directed to the corresponding author.

## Supplementary Materials

The following supporting information can be downloaded at: https://www.mdpi.com/article/10.3390/cells15131228/s1, Supplementary Figure S1: FL-tau fibrils characterization by ThT and tau biosensor assay; Supplementary Figure S2: Time-lapse visualization of tau aggregation in tau biosensor cells; Supplementary Figure S3: PI4KIIIα inhibition with GSK-F1 reduces internalization intracellular levels of FL-tau-488 fibrils when added exogenously to HEK293T cells; Supplementary Figure S4: Targeting strategy for hNGN2 insertion into the AAVS1 locus; Supplementary Figure S5: Quality control of iPS cell lines after integration of inducible hNGN2 cassette; Supplementary Figure S6: Neuronal markers at maturation day 7; Supplementary Figure S7: 4R tau expression over time in NGN2-neurons; Supplementary Figure S8: Neuronal viability after 72 h treatment with PI4KIIIα inhibitors; Supplementary Figure S9: PI4KIIIα inhibition with GSK-F1 moderately reduces internalization intracellular levels of pHrodo-FL-tau seeds when added exogenously to iPS cell-derived neurons; Supplementary Figure S10: Six hours treatment with PI4KIIIα inhibitors does not alter tau aggregation in MAPT-Mut neurons; Supplementary Figure S11: Graph from Figure 2B showing results from individual replicates; Supplementary Figure S12: Graph from Figure 3B showing results from individual replicates; Supplementary Figure S13. Graph from Figure 3D showing results from individual replicates; Supplementary Figure S14. Graph from Figure 4B showing results from individual replicates; Supplementary Figure S15. Graph from Figure 4D showing results from individual replicates; Supplementary Figure S16. Graph from Figure 5D showing results from individual replicates; Supplementary Figure S17. Graph from Figure 6B showing results from individual replicates; Supplementary Figure S18. Graph from Figure 7B showing results from individual replicates; Supplementary Figure S19. Graph from Figure 7C showing results from individual replicates; Supplementary Figure S20. Graph from Figure 7D showing results from individual replicates; Supplementary Figure S21. Graph from Figure 8C showing results from individual replicates; Supplementary Figure S22. Graph from Figure 9B showing results from individual replicates; Supplementary Figure S23. Graph from Figure 9C showing results from individual replicates; Supplementary Figure S24. Graph from Figure S1B showing results from individual replicates; Supplementary Figure S25. Graph from Figure S9B showing results from individual replicates; Supplementary Figure S26. Graph from Figure S9C showing results from individual replicates; Supplementary Figure S27. Graph from Figure S9D showing results from individual replicates; Supplementary Table S1. Primers, siRNAs, and gRNAs; Supplementary materials and methods.

## Abbreviations

The following abbreviations are used in this manuscript:

AAVS1	Adeno-associated virus integration site
CFP	Cyan fluorescent protein
DMSO	Dimethyl sulfoxide
HTRF	Homogeneous Time-Resolved Fluorescence
iPS	Induced pluripotent stem
NGN2	Neurogenin 2
PI4K	Phosphatidylinositol 4-kinase
PI4KIIIα	Phosphatidylinositol 4-kinase III alpha
PI4KIIIβ	Phosphatidylinositol 4-kinase III beta
PI4P	Phosphatidylinositol 4-phosphate
PI(4,5)P2	Phosphatidylinositol 4,5-bisphosphate
WGA	Wheat germ agglutinin
WT	Wild type
YFP	Yellow fluorescent protein
