# Supplementary material for: Reducing PI4KIIIα Levels or Activity Limits Tau Seed Internalization and Assembly in Human Cortical Neurons
Source: Cells. 2026 Jul 7;15(13):1228. doi: 10.3390/cells15131228 (PMC13359555; doi:10.3390/cells15131228)
Supplement: Supplementary file 1 [file cells-15-01228-s001.zip › supplementary info for tau spreading-2026-07-06.pdf]

Supplementary Materials

Title: Reducing PI4KIIIα levels or activity limits tau seed internalization and assembly in human cortical neurons

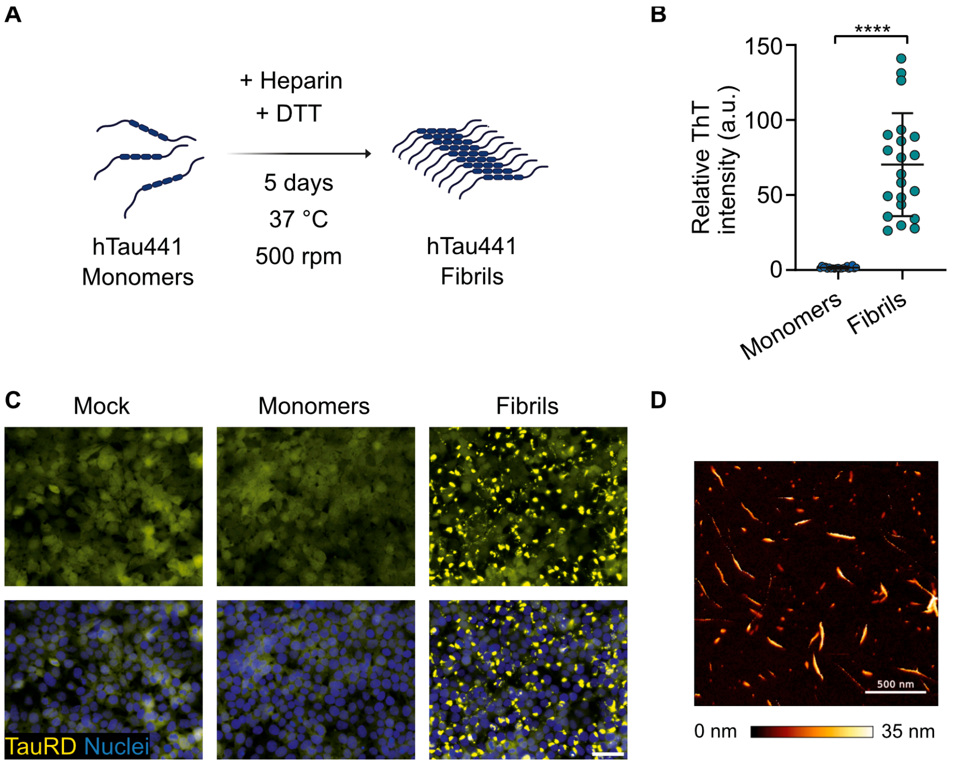

Supplementary Figure S1. FL-tau fibrils characterization by ThT and tau biosensor assay. (A) Diagram showing the fibrillation protocol followed to produce FL-tau fibrils from monomers. Created in BioRender. Sterneckert, J. (2026) https://BioRender.com/qn8rhta. (B) Thioflavin T fluorescence intensity of monomers and fibrils relative to only buffer control after 5 days fibrillation reaction. Data represents individual data points of seven independent fibrillation reactions (N=7) with three technical replicates (n=3) per condition. Error bars represent standard deviation. **** indicates $p<0.0001$ according to two-way ANOVA. See supplementary information for replicate-specific data as well as ANOVA results. (C) Representative fluorescence images of seeded tau biosensor cells with empty liposomes (mock), FL-tau monomers or FL-tau fibrils. Scale bar = 50 µm. Abbreviations: full-length tau (FL-tau), dithiothreitol (DTT), thioflavin T (ThT), revolutions per minute (rpm), arbitrary units (a.u.). (D) Atomic force microscopy topography images of 2N4R FL-tau fibrils adsorbed onto mica substrate.

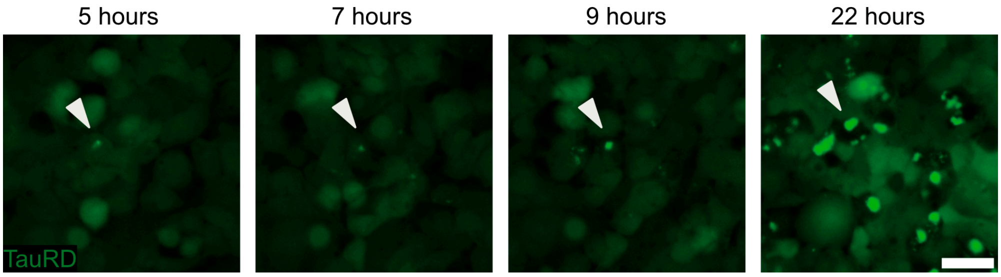

Supplementary Figure S2. Time-lapse visualization of tau aggregation in tau biosensor cells. Fluorescence images acquired with Lumascope 2.0 microscope of tau biosensor cells after transfection of FL-tau seeds. Scale bar = 20 µm.

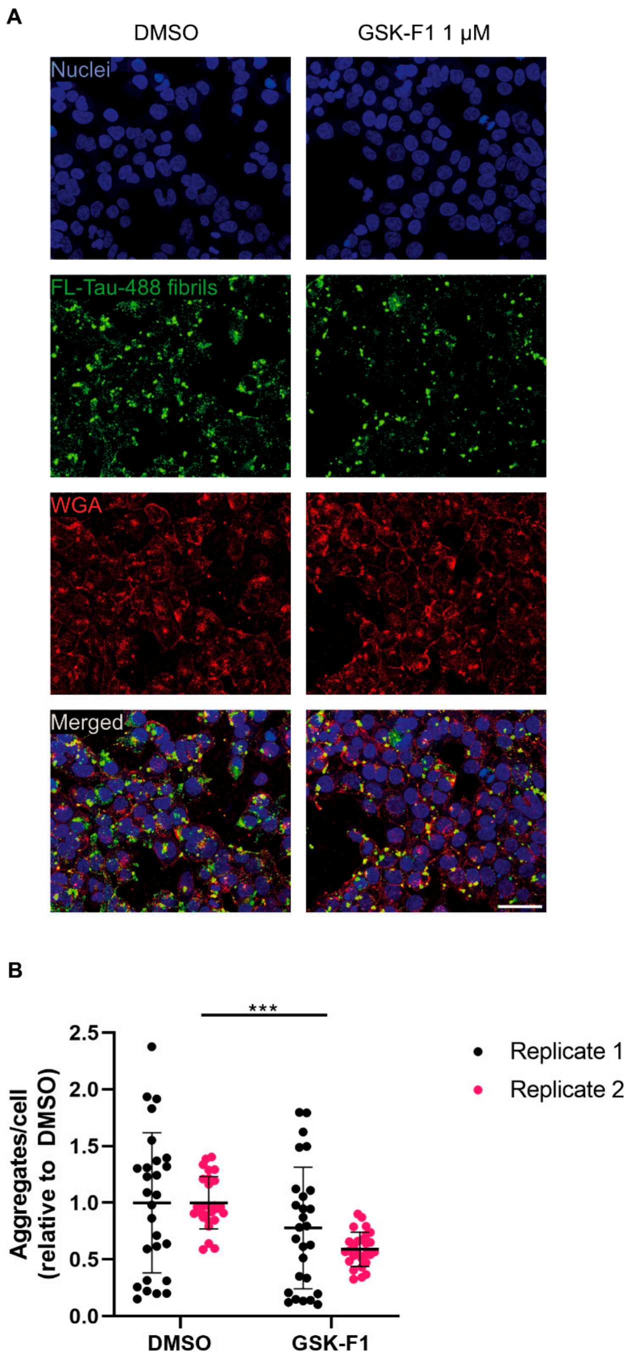

Supplementary Figure S3. PI4KIIIα inhibition with GSK-F1 reduces intracellular levels of FL-tau-488 fibrils when added exogenously to HEK293T cells. (A) Representative fluorescence images of HEK293T cells treated with the indicated concentration of GSK-F1 or the DMSO control. Scale bar = 50 µm. (B) Graph shows mean of all data points of two independent experiments (N=2). Error bars indicate standard deviation. **** indicate $p<0.0001$ according to two-way ANOVA. Since GSK-F1 was diluted from a 10 mM stock solution in DMSO, the DMSO levels in the 1 µM GSK-F1 condition was 0.01% of the total volume. For this reason, the DMSO level in the DMSO control well was also 0.01% of the total volume.

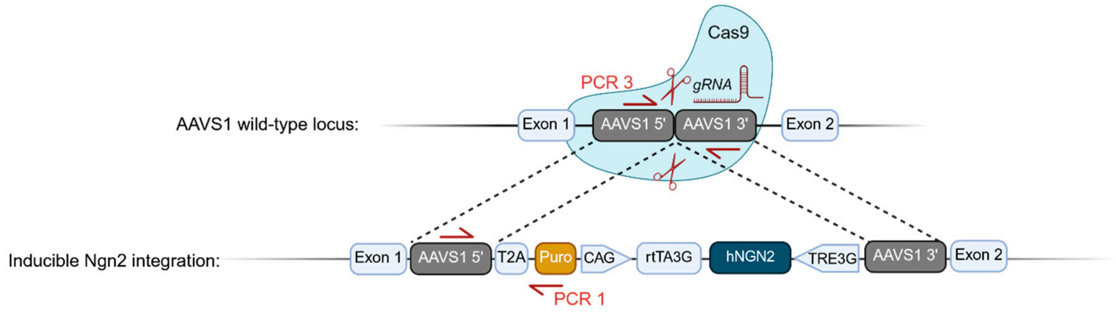

Supplementary Figure S4. Targeting strategy for hNGN2 insertion into the AAVS1 locus. Diagram showing the insertion of the hNGN2 inducible construct inside the AAVS1 locus. PCR1 indicates the primers pair used for detection of the presence of the insert, whereas PCR3 represents the primer pair recognizing only the untargeted locus. Created in BioRender. Sterneckert, J. (2026) https://BioRender.com/x5w2u6h.

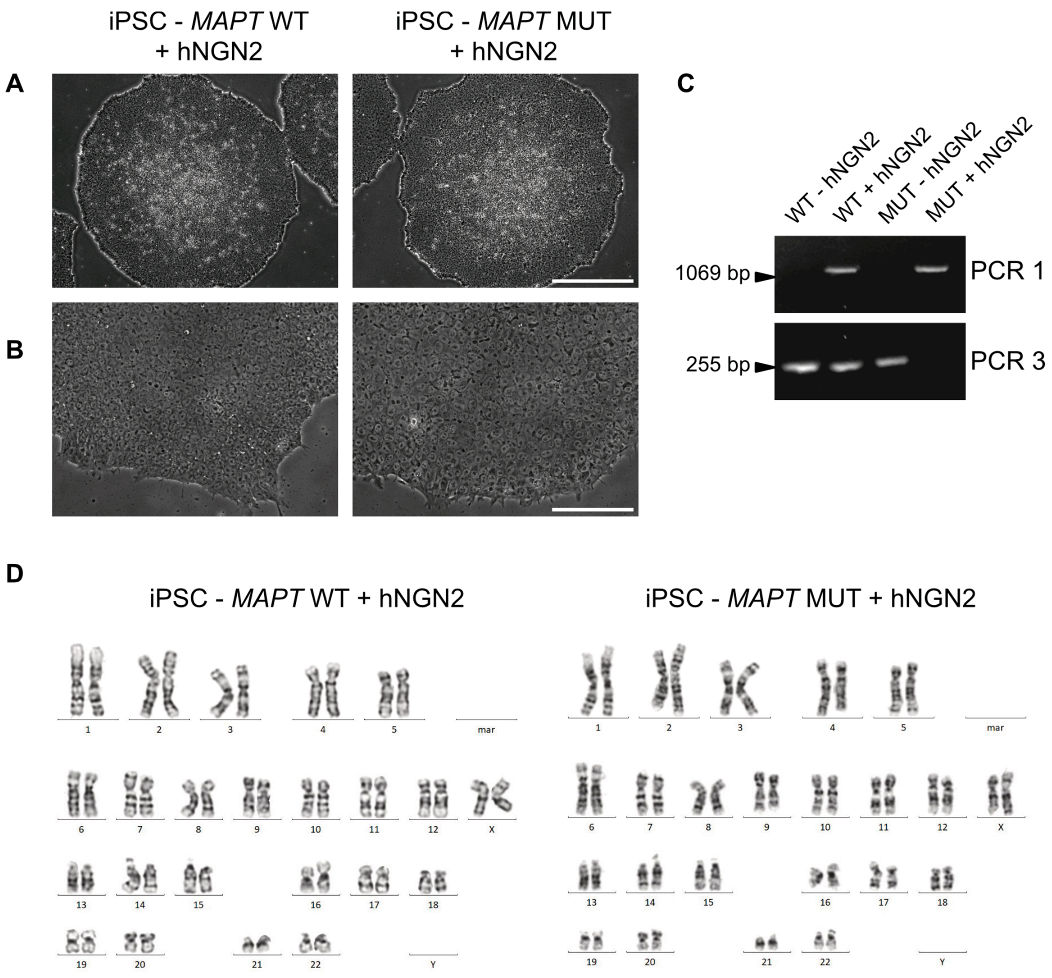

Supplementary Figure S5. Quality control of iPS cell lines after integration of inducible hNGN2 cassette. (A) Brightfield 4x images of MAPT-WT and MAPT-MUT iPS cell colonies, scale bar = 500 µm, or (B) at 20x, scale bar = 100 µm. (C) PCR1 and PCR3 products, before and after editing of MAPT-WT and MAPT-MUT iPS cells. (D) G banding karyotype results showing a normal karyotype for both cell lines.

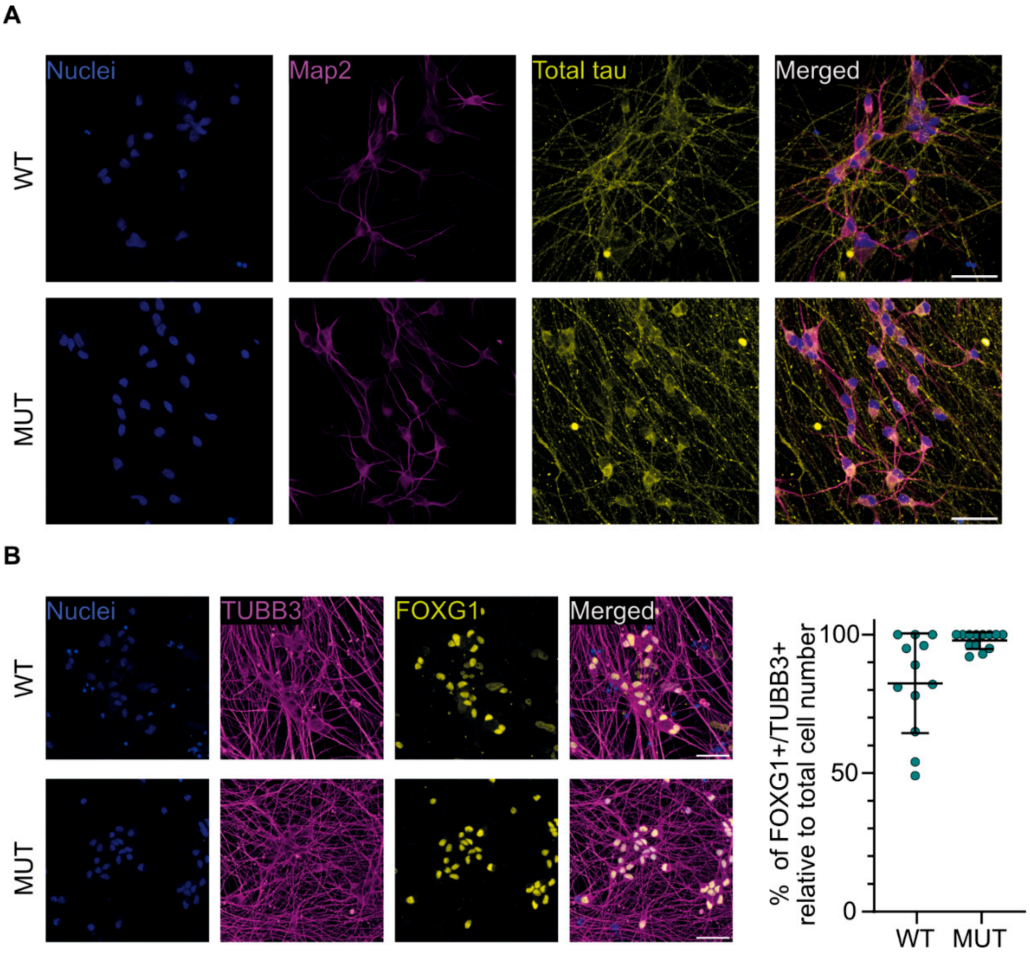

Supplementary Figure S6. Neuronal markers at maturation day 7. (A) Representative immunofluorescence images showing expression of the post-mitotic dendritic marker MAP2 and total tau protein in MAPT-WT and MATP-Mut neurons (B) Representative fluorescence images of MAPT-WT and MAPT-Mut neurons expression of TUBB3 and FOXG1, together with the respective quantification. Quantification represents the percentage of cells expressing both markers in relation to the total cell number. Scale bar = 50 µm.

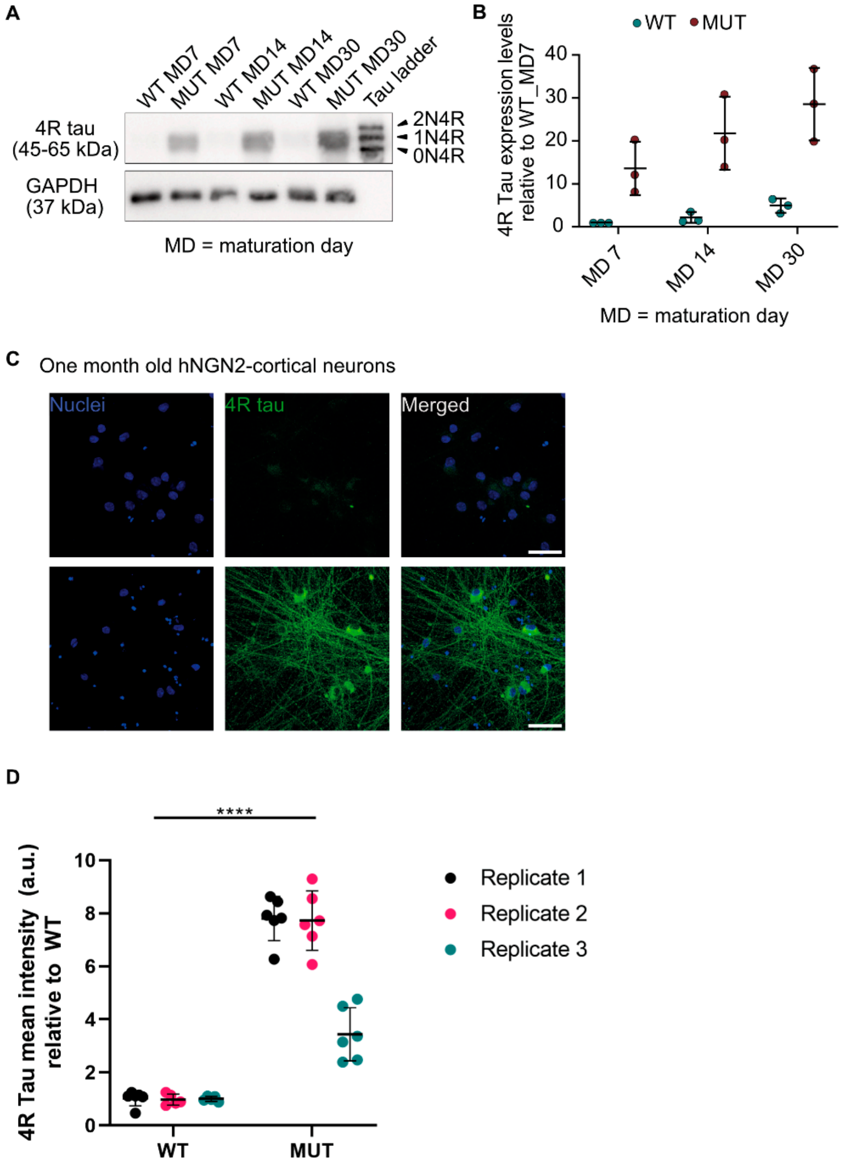

Supplementary Figure S7. 4R tau expression over time in NGN2-neurons. (A) Representative immunoblot of 4R tau and GAPDH protein expression in iPS cell-derived cortical neurons after the indicated number of maturation days. (B) Quantification of 4R tau expression of MAPT-WT and MAPT-Mut neurons relative to the 4R expression level of MAPT-WT neurons at maturation day 7. Graphs show mean of three independent experiments (N=3). (C) 4R tau immunostaining on MAPT-WT and MAPT-Mut neurons at maturation day 30. Scale bar = 50 µm. (D) Quantification of 4R tau immunostaining shows a significant increase of 4R tau expression in MAPT-Mut neurons compared to MAPT-WT neurons after 30 days of maturation. Graph shows mean of all data points of three independent experiments (N=3). Error bars indicate standard deviation. **** indicates $p<0.0001$ according to two was ANOVA. Abbreviations: maturation day (MD). See supplementary information for replicate-specific data as well as ANOVA results.

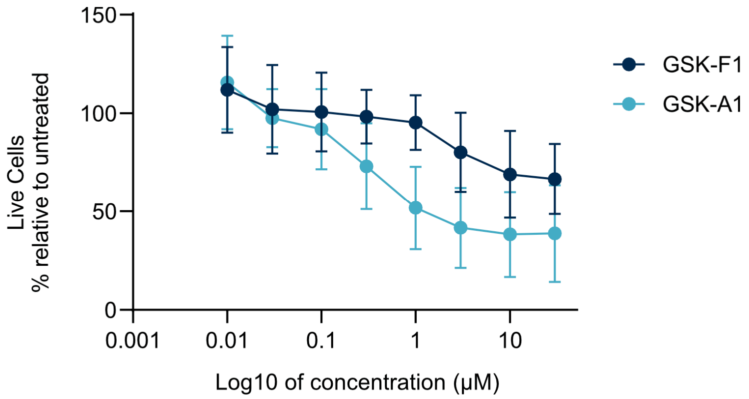

Supplementary Figure S8. Neuronal viability after 72 hours treatment with PI4KIIIα inhibitors. Percentage of viable neurons relative to the untreated control after treatment with different concentrations of the PI4KIIIα inhibitors GSK-F1 (dark blue) and GSK-A1 (light blue). Results show average viability of four independent experiments (N=4). Error bars represent standard deviation. IC_{50} for GSK-F1 is 2.192 μM and for GSK-A1 is 0.244 μM, calculated by non-linear regression using GraphPad Prism 8.

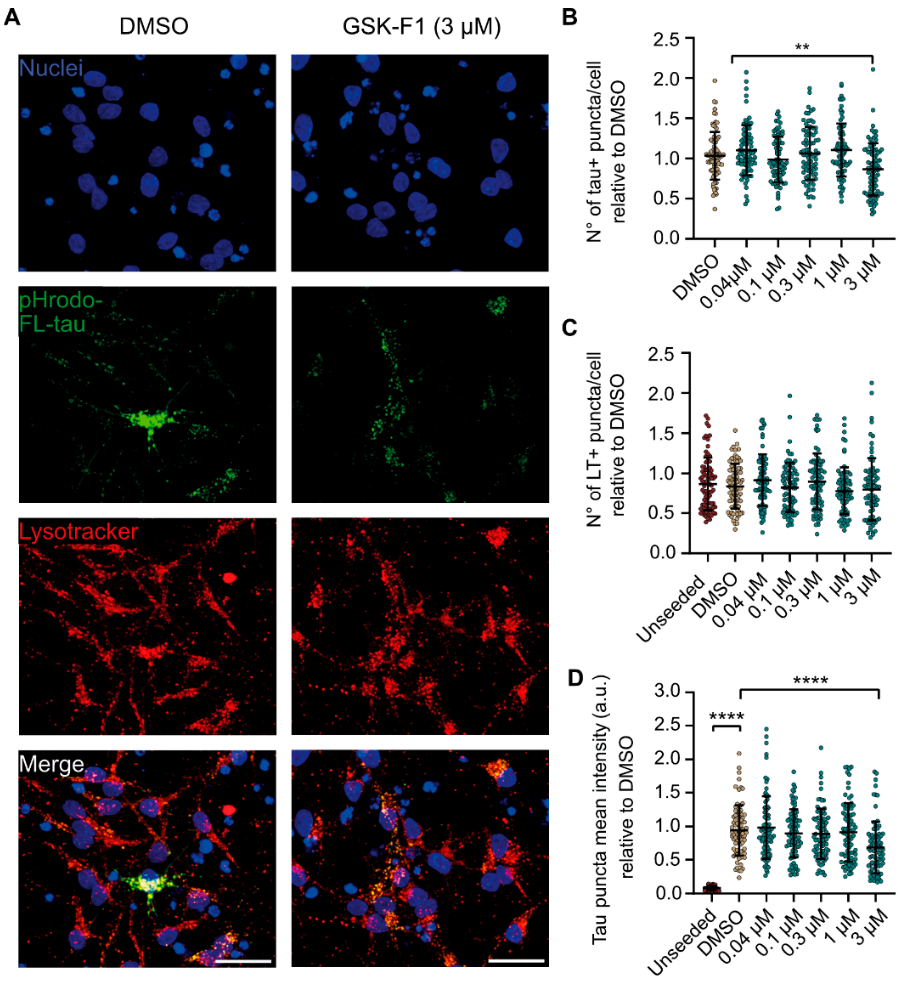

Supplementary Figure S9. PI4KIIIα inhibition with GSK-F1 moderately reduces intracellular levels of pHrodo-FL-tau seeds when added exogenously to iPS cell-derived neurons. (A) Representative fluorescence images of MAPT-Mut neurons on maturation day 7 treated with the indicated concentration of GSK-F1 or the DMSO control and pHrodo-FL-tau seeds. Scale bar = 50 μm. (B-D) Graphs showing the mean of all data points of three independent experiments (N=3). Error bars indicate standard deviation. ****, *** and ** indicate $p<0.0001$, $p<0.001$ and $p<0.01$, respectively, according to two-way ANOVA followed by Dunnett's multiple comparisons test against the DMSO control. Abbreviations: Lysotracker (LT). Since GSK-F1 was diluted from a 10 mM stock solution in DMSO, the DMSO levels in the 3 μM condition was 0.03 % of the total volume. For this reason, the DMSO level in the DMSO control well was also 0.03% of the total volume. See supplementary information for replicate-specific data as well as ANOVA results.

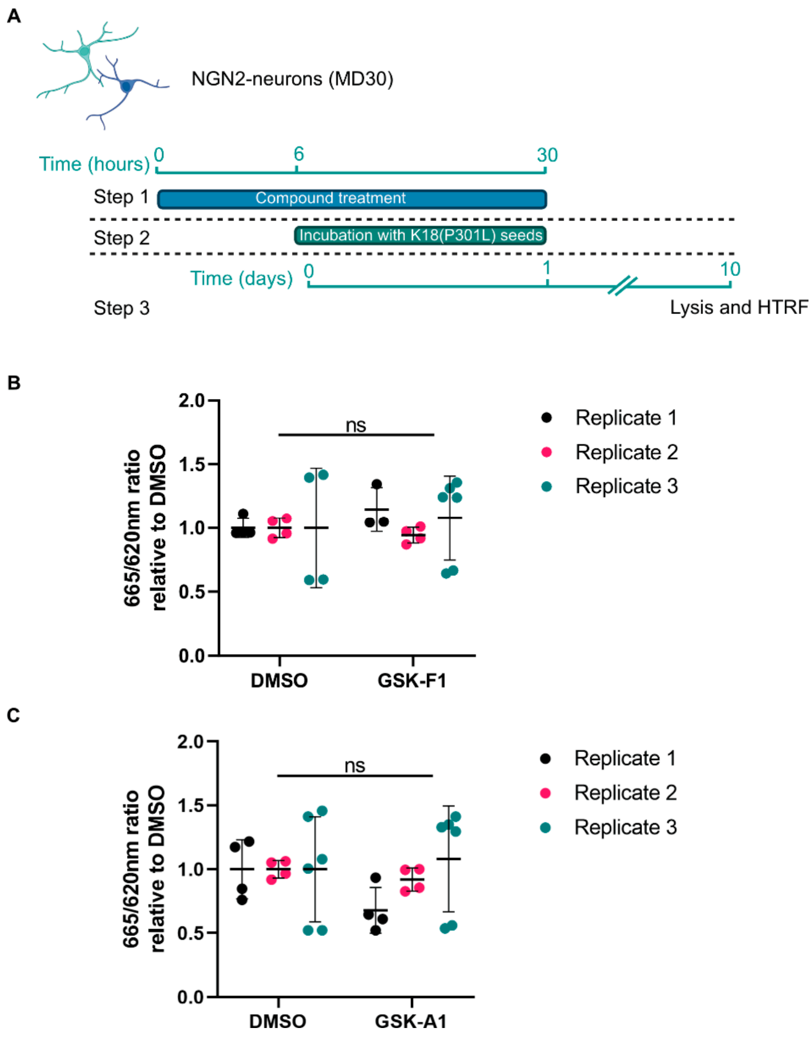

Supplementary Figure S10. Six hours treatment with PI4KIIIα inhibitors does not alter tau aggregation in MAPT-Mut neurons. (A) Diagram illustrating the experimental design of the tau aggregation assay, in which neurons were treated for 24 hours with K18(P301L)-tau seeds and lysis for HTRF was performed 10 days after addition of the seeds. Compounds were added 6 hours prior to the addition of the seeds and were present on the media during the total duration of the treatment with K18(P301L)-tau seeds. Tau aggregation HTRF in 30 days mature MAPT-Mut neurons treated with the indicated concentrations of (B) GSK-F1 or (C) GSK-A1. Created in BioRender. Sterneckert, J. (2026) https://BioRender.com/2o0a2ks. Graphs show mean of all data point of three independent experiments (N=3). Error bars represent standard deviation. Not significant (ns) according to two-way ANOVA. Abbreviations: Maturation day (MD). See supplementary information for replicate-specific data as well as ANOVA results.

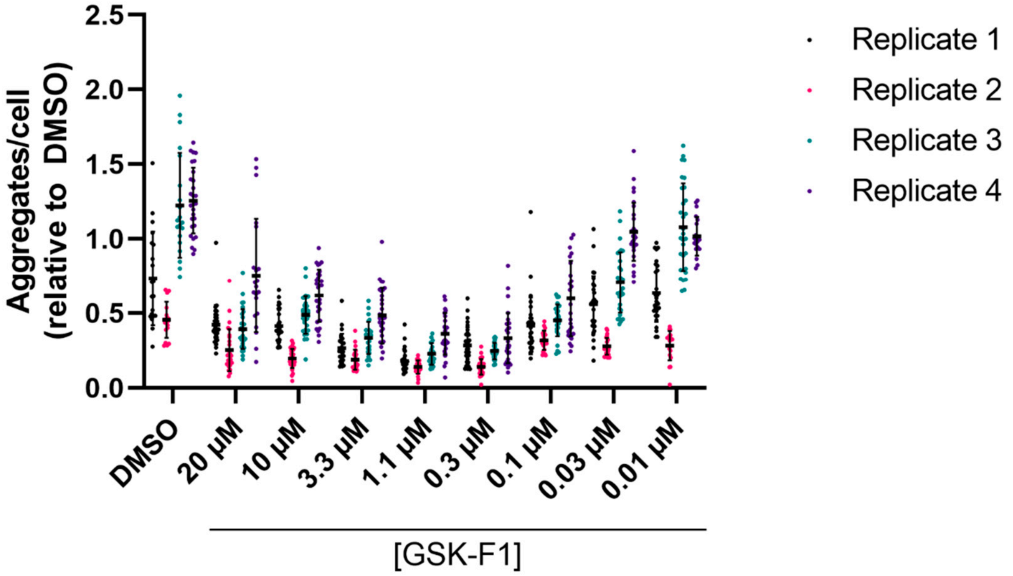

Supplementary Figure S11. Graph from Figure 2B showing results from individual replicates. Details about statistical significance calculations can be found in supplementary excel file.

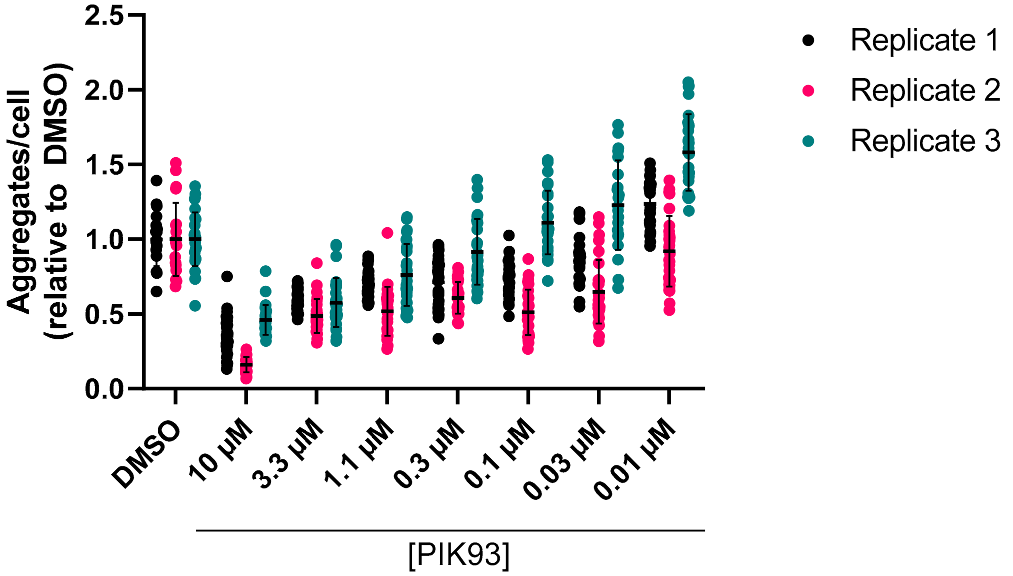

Supplementary Figure S12. Graph from Figure 3B showing results from individual replicates. Details about statistical significance calculations can be found in supplementary excel file.

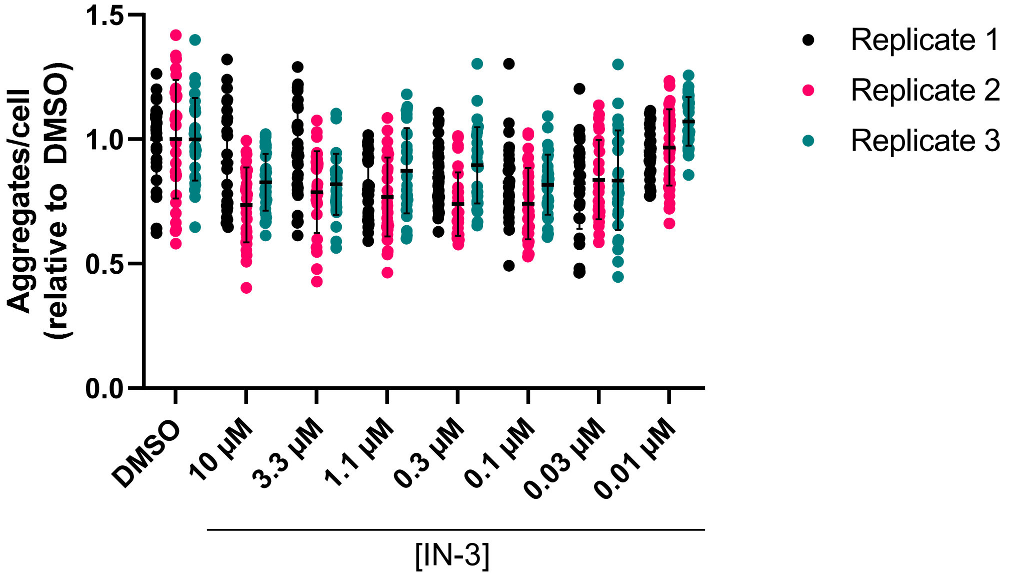

Supplementary Figure S13. Graph from Figure 3D showing results from individual replicates. Details about statistical significance calculations can be found in supplementary excel file.

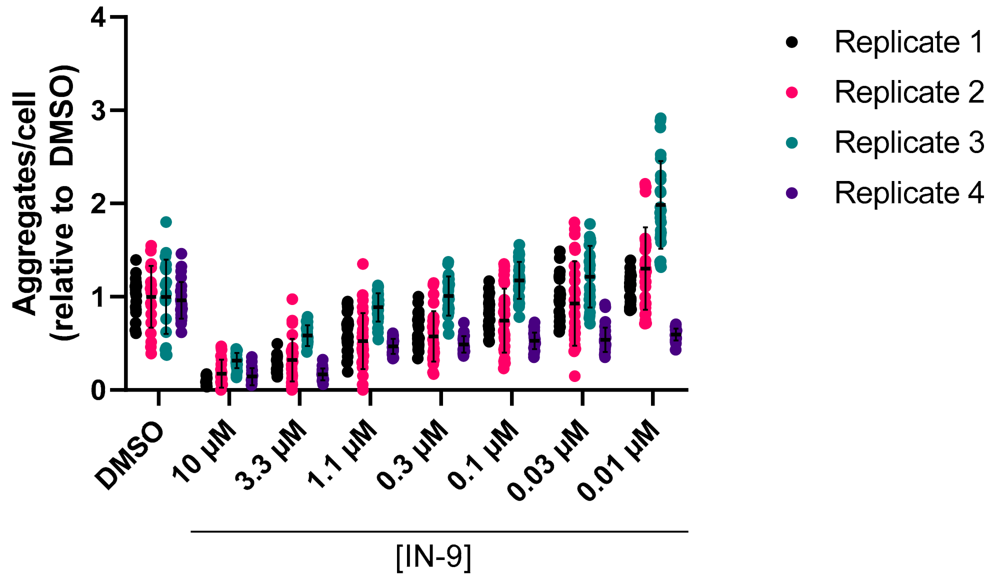

Supplementary Figure S14. Graph from Figure 4B showing results from individual replicates. Details about statistical significance calculations can be found in supplementary excel file.

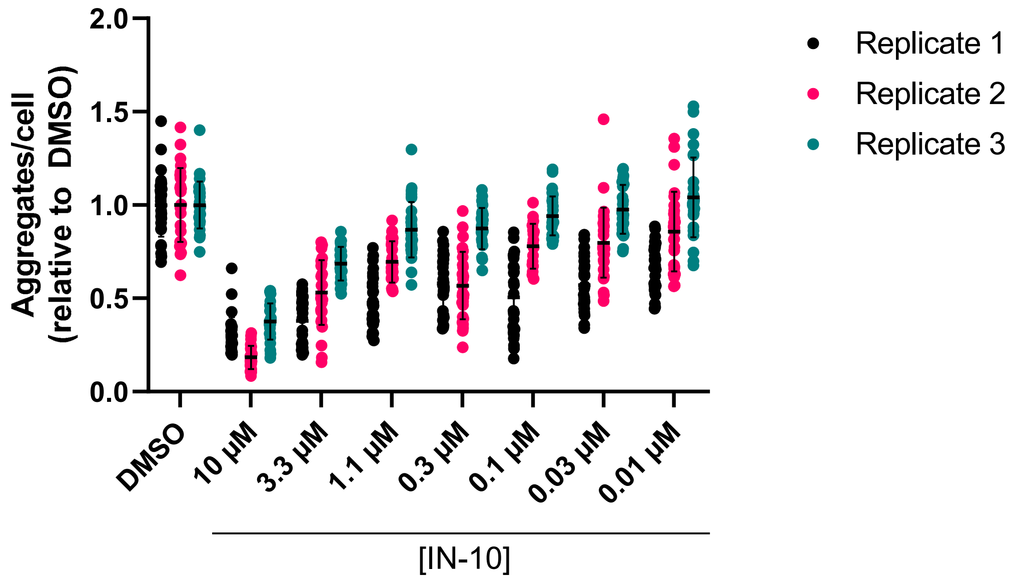

Supplementary Figure S15. Graph from Figure 4D showing results from individual replicates. Details about statistical significance calculations can be found in supplementary excel file.

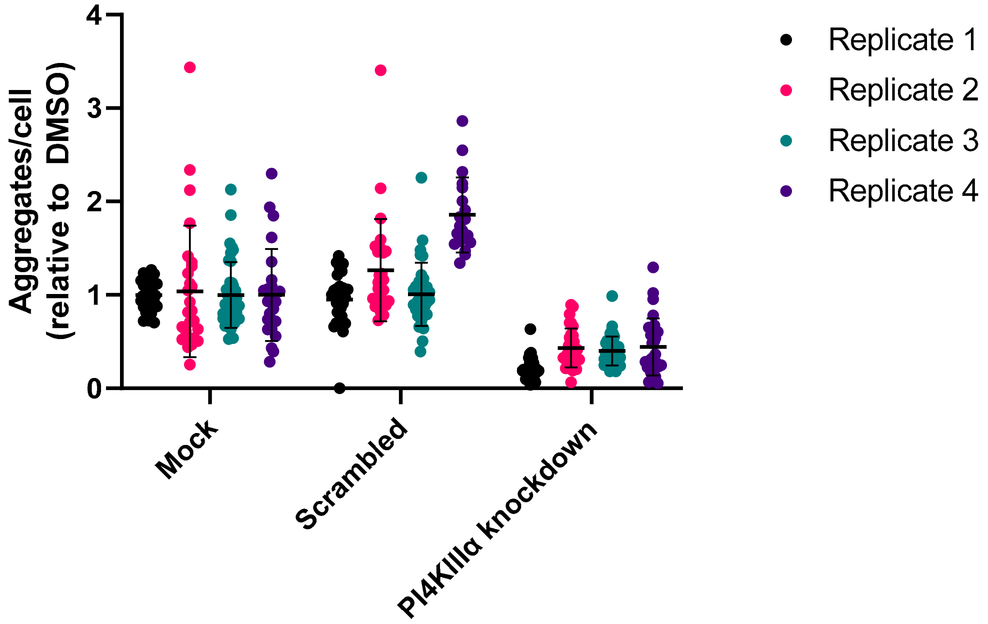

Supplementary Figure S16. Graph from Figure 5D showing results from individual replicates. Details about statistical significance calculations can be found in supplementary excel file.

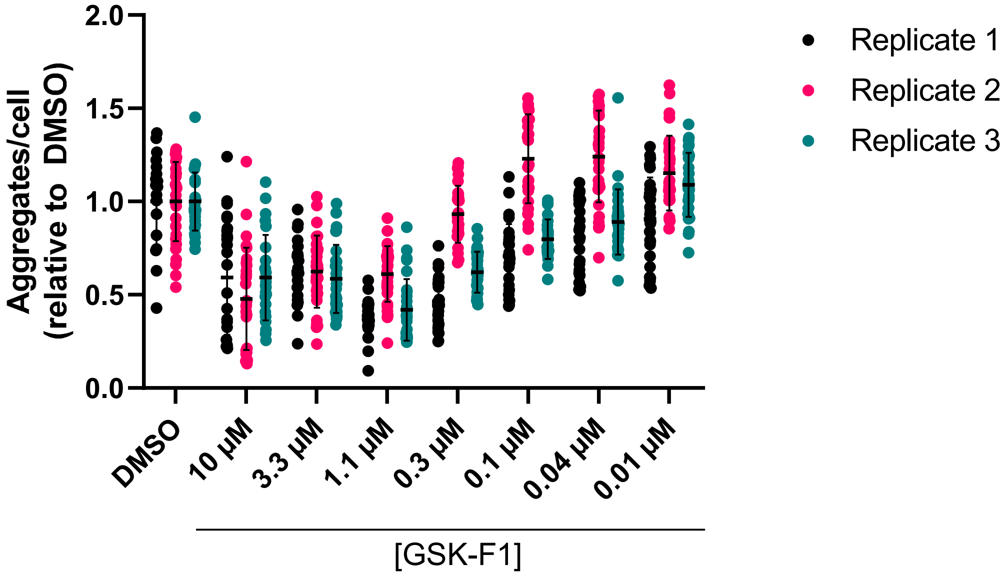

Supplementary Figure S17. Graph from Figure 6B showing results from individual replicates. Details about statistical significance calculations can be found in supplementary excel file.

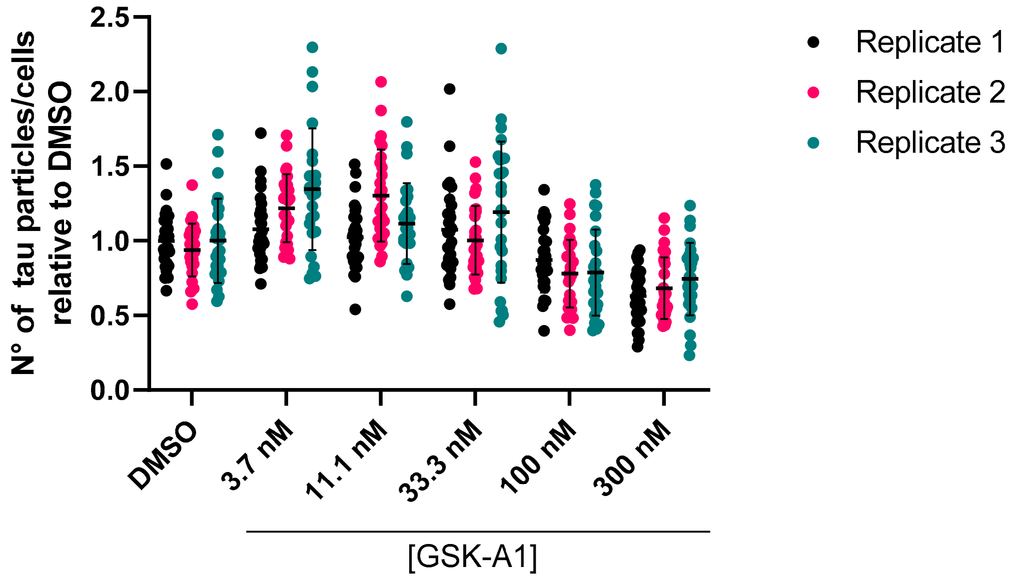

Supplementary Figure S18. Graph from Figure 7B showing results from individual replicates. Details about statistical significance calculations can be found in supplementary excel file.

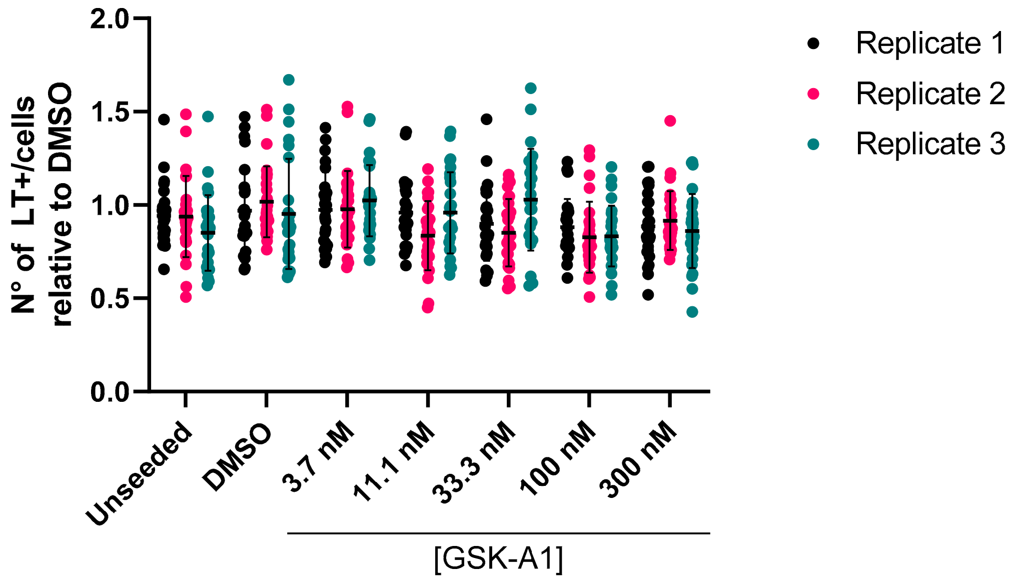

Supplementary Figure S19. Graph from Figure 7C showing results from individual replicates. Details about statistical significance calculations can be found in supplementary excel file.

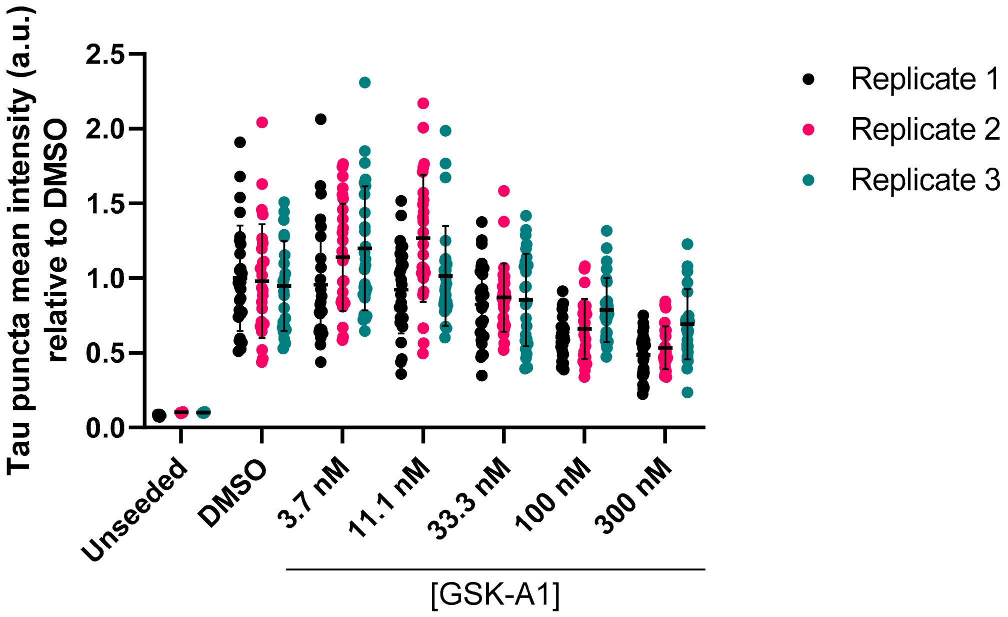

Supplementary Figure S20. Graph from Figure 7D showing results from individual replicates. Details about statistical significance calculations can be found in supplementary excel file.

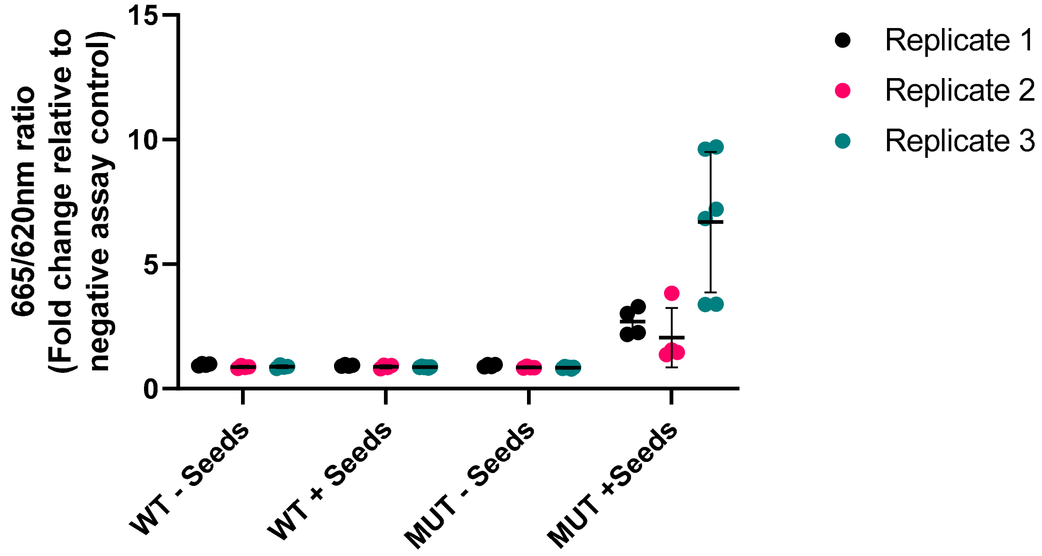

Supplementary Figure S21. Graph from Figure 8C showing results from individual replicates. Details about statistical significance calculations can be found in supplementary excel file.

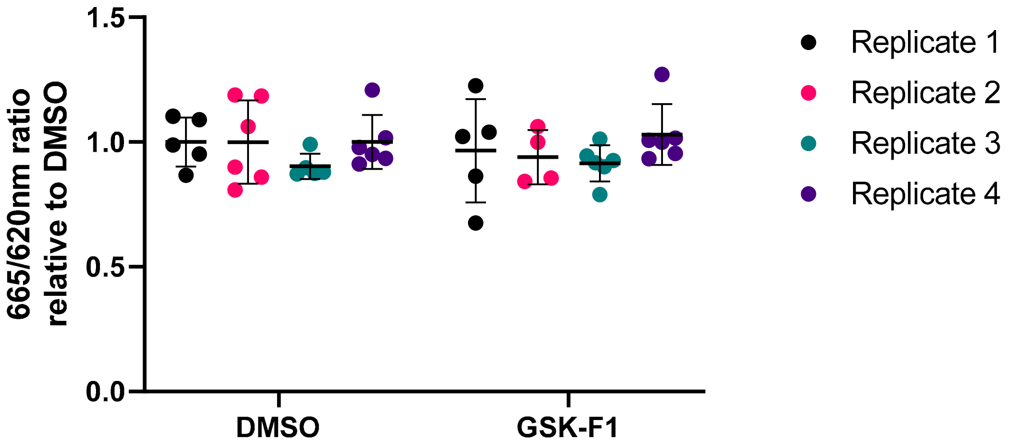

Supplementary Figure S22. Graph from Figure 9B showing results from individual replicates. Details about statistical significance calculations can be found in supplementary excel file.

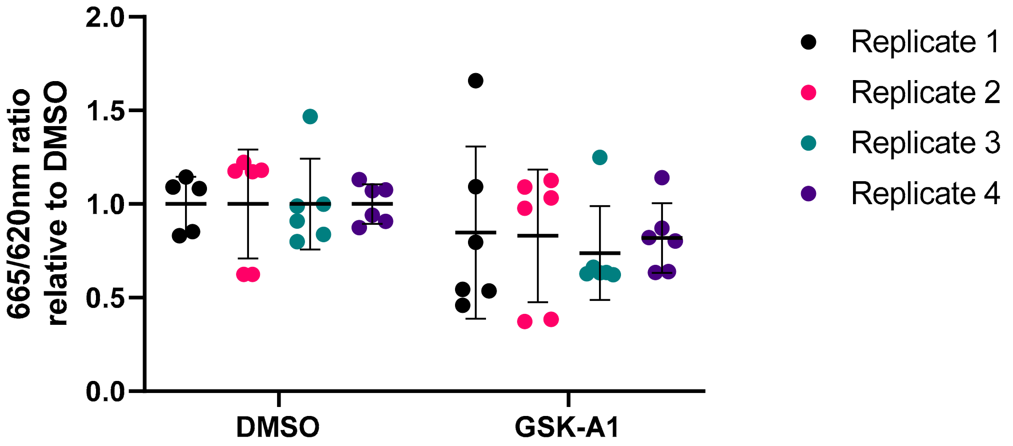

Supplementary Figure S23. Graph from Figure 9C showing results from individual replicates. Details about statistical significance calculations can be found in supplementary excel file.

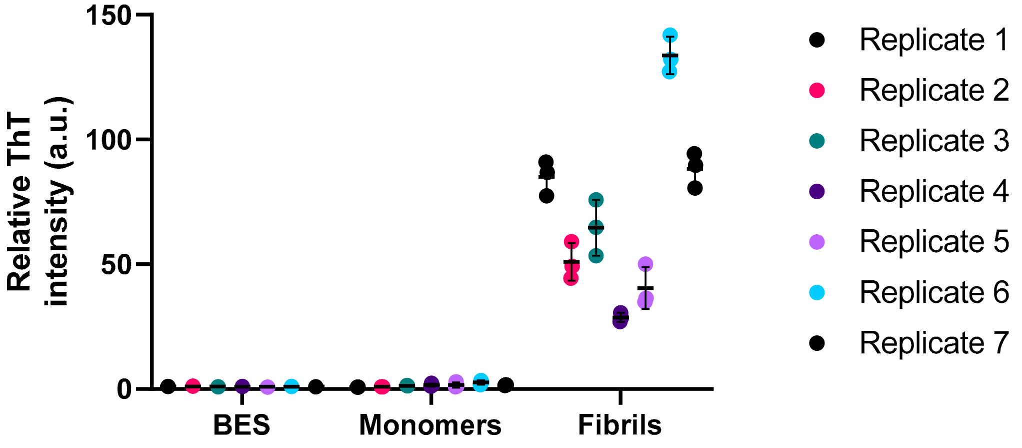

Supplementary Figure S24. Graph from Figure S1B showing results from individual replicates. Details about statistical significance calculations can be found in supplementary excel file.

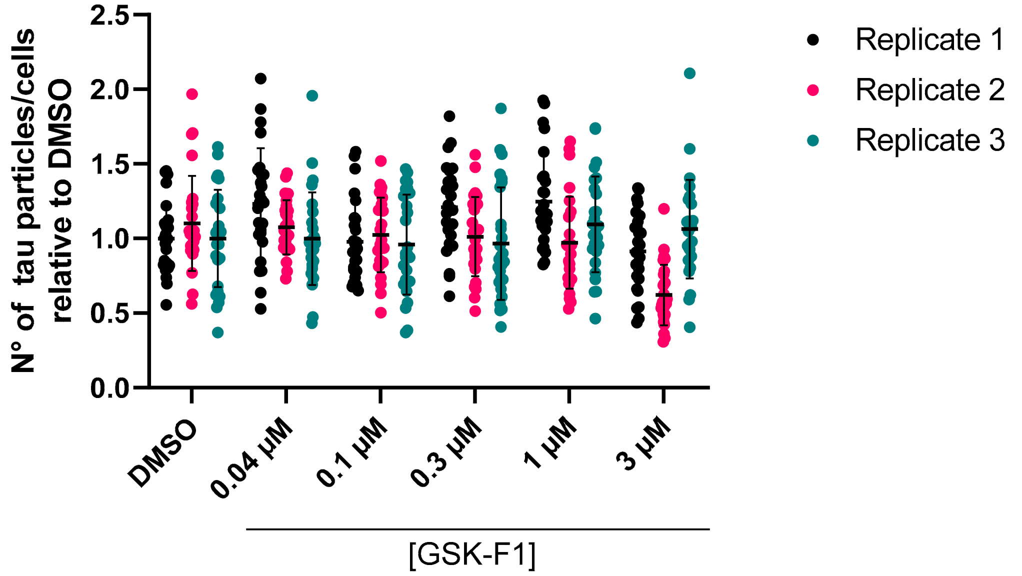

Supplementary Figure S25. Graph from Figure S9B showing results from individual replicates. Details about statistical significance calculations can be found in supplementary excel file.

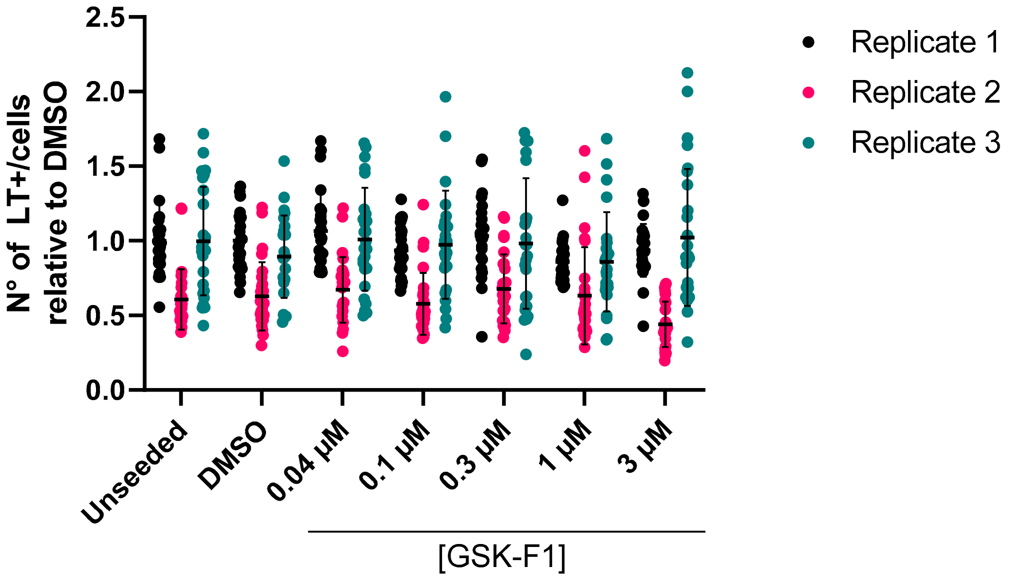

Supplementary Figure S26. Graph from Figure S9C showing results from individual replicates. Details about statistical significance calculations can be found in supplementary excel file.

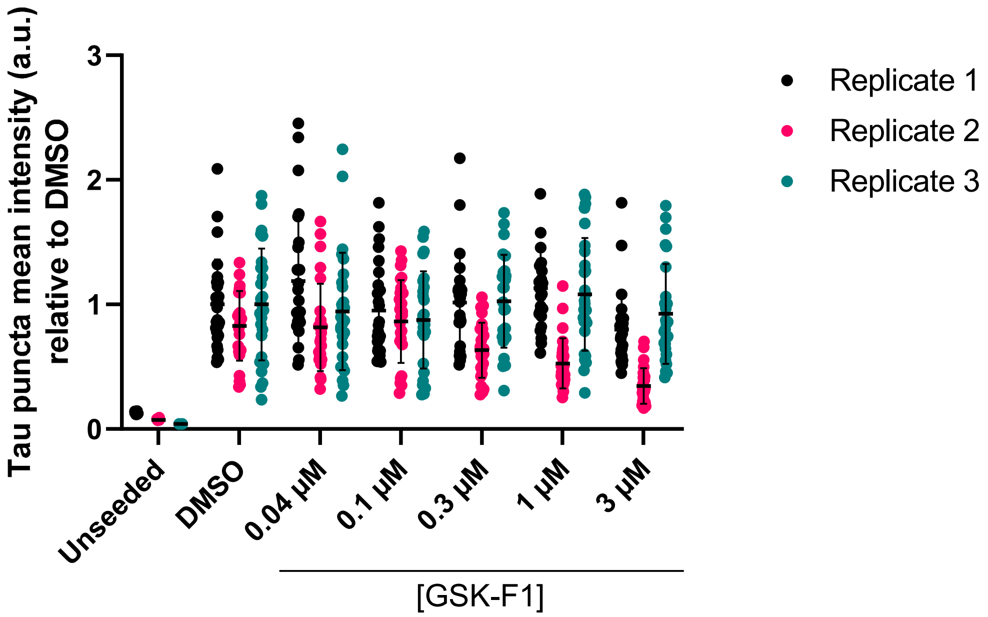

Supplementary Figure S27. Graph from Figure S9D showing results from individual replicates. Details about statistical significance calculations can be found in supplementary excel file.

Supplementary Materials and Methods

Fibrillation reactions and thioflavin T assay

Tau monomers were mixed to a 20 µM final concentration in BES buffer supplemented with 1 mM DTT. Samples were incubated at 75 °C in a Thermomixer to break intra-disulfide bridges. After cooling down the samples, heparin was added to a 5 µM final concentration to the tau samples. The tau-to-heparin-molar ratio must always be 4:1. Tau samples were incubated in a Thermomixer at 37 °C and shaking at 500 rpm for 24 hours in the case of K18 and K18(P301L) or for 5 days to obtain full length 2N4R tau seeds. Working aliquots were stored at -80°C until used.

For α-synuclein pre-formed fibrils production, a ready to use solution was obtained from Proteos. The stock solution was centrifuged for 10 minutes at 12,000 rpm on a bench centrifuge. The 10 mg/ml α-synuclein monomer solution was diluted in DPBS to a final concentration of 5mg/ml and incubated for 3 days in an orbital shaker at 1000 rpm and 37 °C. Fibril formation was verified by thioflavin T assay. Working aliquots were stored at -80 °C until used.

Fibrillation was verified for every batch of tau seeds or α-synuclein seeds by Thioflavin T (ThT) assay. For the ThT assay, 7.5 µM of final protein concentration (fibrils, monomers or buffer control) were incubated with 25 µM ThT, in BES buffer for tau or DPBS for α-synuclein. Samples were mixed in 384-well plates, incubated for 1 hour at room temperature and protected from light. Endpoint fluorescence was measured using a Biotek Synergy Neo plate reader by exciting the samples at 448 nm and measuring emission at 482 nm. Fold change was calculated by dividing all values by the average diluent/buffer signal.

Tau seeds fluorescent labeling

Full length tau seeds were labeled with DyLight™ 488 or pHrodo™ 488 according to manufacturer's instructions. Briefly, initial protein concentration was estimated by BCA assay and 1 µg/µl of protein was incubated with the labelling reagent for 1 hour at room temperature in the dark. After labeling, excess dye was removed using dialysis. Protein samples were transferred into dialysis columns (D-Tube™ dialyzers Midi) with a molecular weight cut-off of 12–14 kDa. The columns were first incubated with 200 µl of distilled H_2O, which was removed prior to the addition of the labelled tau seeds. The column was then immersed in at least 10-fold excess volume of dialysis buffer (DPBS). To keep the columns upright a floating rack was used. The entire setup was placed inside a sterile bottle to maintain aseptic conditions. A magnetic stir bar was added to the buffer, and the bottle was positioned on a magnetic stirrer to ensure continuous mixing throughout the dialysis process. Dialysis was performed overnight at 4 °C and samples were protected from light. The following morning, dialyzed protein samples were retrieved from the columns using a pipette and transferred into fresh sterile tubes. Labeled proteins were stored at 4 °C until used.

Multiwell plates coating procedures

To prepare gelatin-coated plates, a sufficient volume of gelatin solution was added to each well to completely cover the surface. The plates were incubated at room temperature for 2-3 hours to allow proper coating. Immediately prior to cell seeding, the gelatin solution was removed by vacuum aspiration.

Cultrex-coated plates were prepared by diluting Cultrex-SC (1:100) or Cultrex-RGF (1:200) in DMEM/F-12 medium. Stock vials of Cultrex were thawed on ice for at least 1 hour prior to use. The diluted solution was added to each well at a volume sufficient to fully cover the surface. Plates were

then wrapped with Parafilm and incubated overnight at room temperature to allow matrix polymerization. Following incubation, coated plates were stored at 4 °C and used within two weeks. Cultrex-SC was used to culture iPS cells, whereas Cultrex-RGF was used for neuronal induction and differentiation.

Neurons undergoing maturation were cultured on plates coated with PLO/Laminin-521. For coating, wells were first treated with a 30% PLO solution in DPBS and incubated overnight at 37 °C. The following day, the PLO solution was recovered and reused for up to three coating cycles. Wells were then washed three times with sterile water and allowed to air dry completely. Laminin-521 was then diluted 1:20 in DPBS supplemented with 0.5 mM $MgCl_2$ and 0.9 mM $CaCl_2$, and the diluted solution was added to the PLO-coated wells. Plates were incubated overnight at 4 °C and stored under the same conditions for up to one week prior to use.

Plasmid DNA isolation

pXAT2 was a gift from Knut Woltjen (Addgene plasmid #80494; http://n2t.net/addgene:80494; RRID:Addgene_80494). pUCM-AAVS1-TO-hNGN2 was a gift from Michael Ward (Addgene plasmid #105840; http://n2t.net/addgene:105840; RRID:Addgene_105840). The gRNA used for targeting the AAVS1 locus contained in the pXAT2 plasmid is the following: 5'-GGGGCCACTAGGGACAGGAT-3' (Oceguera-Yanez et al. 2016).

A primary culture was prepared from the purchased stab culture in 3 ml LB medium (in house supply) supplemented with the required antibiotic (Ampicillin, 1 mg/ml) and incubated at 37 °C for 8 hours. The primary culture was then transferred to a secondary culture in 300 ml LB medium supplemented with the same concentration of Ampicillin and incubated at 37 °C overnight in an orbital shaker. The following morning, cultures were collected and the bacteria were ultracentrifuged for 15 minutes at 4 °C and 6000 x*g*. The pellet was resuspended in the appropriate buffer required by the NucleoBond Xtra Maxi kit and further steps were performed according to manufacturer's instructions. The final pellet was resuspended in 200 µl of nuclease-free water and pDNA concentration was estimated using a NanoDrop™ 2000.

Genomic DNA isolation and Polymerase Chain Reaction (PCR)

To asses insertion of the NGN2 doxycycline-inducible cassette into MAPT-WT and MAPT-Mut iPS cell lines, we isolated genomic DNA from one 80% confluent well of a 6-well multiwell plate. Cells were washed three times with DPBS and lysis buffer was added directly to the well. After collecting the lysate with a scraper, DNA extraction was performed according to manufacturer's instructions for the DNeasy blood and tissue kit (Qiagen). The DNA concentration was determined using a NanoDrop™ 2000 (ThermoFischer Scientific, ND2000). DNA was stored at -20 °C until the PCR was performed.

For the amplification of the DNA fragments of interest, we used a Q5® High-Fidelity Polymerase, and forward and reverse primers flanking the AAVS1 locus (table 3). A negative control without the DNA template was included to detect possible DNA contaminations. The final reaction mix was composed of 100 ng of the DNA template (or water for the negative control), 200 µM deoxynucleotide (dNTP) solution mix, 0.5 µM forward primer, 0.5 µM reverse primer. 1x Q5® high GC enhancer and 0.02 units/µl or Q5® High-Fidelity DNA Polymerase. The reaction conditions used were the following: initial denaturation for 30 seconds at 98°C, followed by 30 cycles of denaturation at 98°C for 15 seconds, annealing at the required melting temperature (Tm) or 25 seconds and extension at 72 °C. A final

extension cycle of 2 minutes at 72 °C was performed to terminate the reaction. Tm was calculated using the New England Biolabs website: https://tmcalculator.neb.com/. Part of the PCR product was loaded on a 1.5% agarose gel to separate and visualize the amplified DNA.

Name	Sequence	Citation
PCR1	Forward: CTGCCGTCTCTCTCCTGAGT<br>Reverse: GGGCTTGTACTCGGTCATCT	[16]
PCR3	Forward: CGGTTAATGTGGCTCTGGTT<br>Reverse: AGGATCCTCTCTGGCTCCAT	[16]
gRNA targeting AAVS1	GGGGCCACTAGGGACAGGAT	[17]
Non-targeting siRNA #3	UGGUUUACAUGUUUUCUGA	Dharmacon Ref. D-001810-03-05
On target siRNA #14 PI4KA	GAUCGAGCGUCUCAUCACA	Dharmacon Ref. J-006776-14

Supplementary Table S1. Primers, siRNAs, and gRNAs.
